# Ligand Control of ^59^Co Nuclear Spin Relaxation Thermometry

**DOI:** 10.3390/magnetochemistry6040058

**Published:** 2020-11-12

**Authors:** Tyler M. Ozvat, Spencer H. Johnson, Anthony K. Rappé, Joseph M. Zadrozny

**Affiliations:** Department of Chemistry, Colorado State University, 1301 Center Ave., Fort Collins, CO 80523-1872, USA

**Keywords:** cobalt-59 NMR, magnetic relaxation, nuclear spins, quadrupolar interaction

## Abstract

Studying the correlation between temperature-driven molecular structure and nuclear spin dynamics is essential to understanding fundamental design principles for thermometric nuclear magnetic resonance spin-based probes. Herein, we study the impact of progressively encapsulating ligands on temperature-dependent ^59^Co *T*_1_ (spin–lattice) and *T*_2_ (spin–spin) relaxation times in a set of Co(III) complexes: K_3_[Co(CN)_6_] (**1**); [Co(NH_3_)_6_]Cl_3_ (**2**); [Co(en)_3_]Cl_3_ (**3**), en = ethylenediamine); [Co(tn)_3_]Cl_3_ (**4**), tn = trimethylenediamine); [Co(tame)_2_]Cl_3_ (**5**), tame = triaminomethylethane); and [Co(dinosar)]Cl_3_ (**6**), dinosar = dinitrosarcophagine). Measurements indicate that ^59^Co *T*_1_ and *T*_2_ increase with temperature for **1**–**6** between 10 and 60 °C, with the greatest Δ*T*_1_/Δ*T* and Δ*T*_2_/Δ*T* temperature sensitivities found for **4** and **3**, 5.3(3)%*T*_1_/°C and 6(1)%*T*_2_/°C, respectively. Temperature-dependent *T*_2_* (dephasing time) analyses were also made, revealing the highest Δ*T*_2_*/Δ*T* sensitivities in structures of greatest encapsulation, as high as 4.64%*T*_2_*/°C for **6**. Calculations of the temperature-dependent quadrupolar coupling parameter, Δ*e*^2^*qQ*/Δ*T,* enable insight into the origins of the relative Δ*T*_1_/Δ*T* values. These results suggest tunable quadrupolar coupling interactions as novel design principles for enhancing temperature sensitivity in nuclear spin-based probes.

## 1. Introduction

The control of nuclear spin properties by molecular design is an important capability for many applications, spanning from diagnostic bioimaging [1–3] to encoding and processing quantum information [4–7]. A more focused application is designing temperature dependence into nuclear spin properties toward molecular-level thermometry, an essential technique for next-generation treatments of cancer [8–11]. Here, ^59^Co nuclear spins are an extremely promising platform for detecting changes in temperature, owing to the extreme thermal sensitivity of the metal ion chemical shift [12]. We note that chemical shift is not the only temperature-dependent property of nuclear spins. Indeed, the influence of temperature on nuclear spin relaxation dynamics may provide a practical additional mechanism for thermometry. Importantly, the quadrupolar coupling of the ^59^Co (*I* = ^7^/_2_) nucleus is exquisitely sensitive to subtle changes in the structure of the coordination shell. Thus, slight temperature-dependent structural changes are expected to drive nuclear spin behaviors by manipulating the quadrupolar coupling interaction, inducing temperature dependence in the ^59^Co spin–lattice and spin–spin relaxation times, *T*_1_ and *T*_2_, respectively. We note that other, more common nuclear spin-based probes, e.g., ^1^H, ^13^C, ^19^F, and ^31^P, are all *I* = ^1^/_2_, are not quadrupolar nuclei, and thus do not sense changes in temperature in this manner [13–15].

Owing to the foregoing advantages, we target design strategies to control the temperature sensitivity of ^59^Co nuclear spin dynamics in encapsulating ligands, which can prevent chemical decomposition in vivo, avoiding the release of toxic metal-ions [16–18]. Recent work by us demonstrated that the interconnected structures of encapsulating scaffolds amplify temperature sensitivity for contained ^59^Co nuclei [19]. Importantly, these studies probed only temperature-driven changes in chemical shift. In contrast, it is unknown to what extent, if any, encapsulation affects the temperature dependence of ^59^Co nuclear spin relaxation processes.

**Figure 1.**
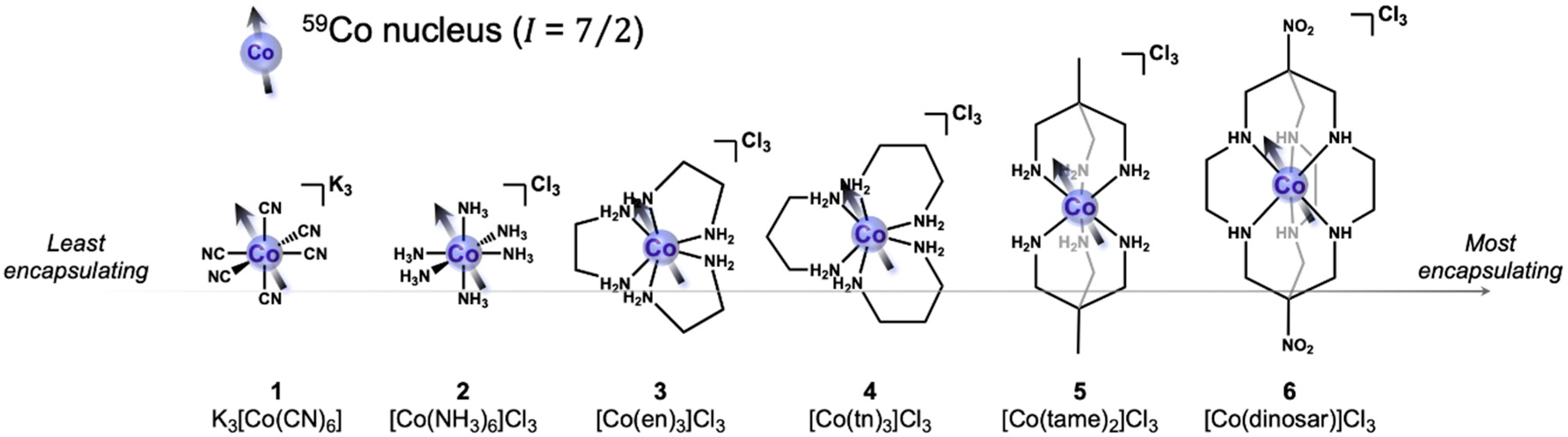
Chemical structure series of low-spin octahedral cobalt(III) complexes. Complexes **2–6** make up the series of progressively encapsulated ^59^Co nuclei by greater degrees of chelation in a common Co–N_6_ coordination environment. Arrows represent the *I* = ^7^/_2_ nuclear spin of the ^59^Co nuclei in each complex. Hydrogens bound to carbons are omitted for clarity.

Herein, we provide the first test of the effect of encapsulation on the thermometric capabilities of the ^59^Co nuclear spin dynamics in Co(III) complexes. To do so, we performed variable-temperature ^59^Co NMR relaxation time experiments, specifically *T*_1_, *T*_2_, and linewidth analysis (*T*_2_*) with a series of six octahedral and pseudo-octahedral cobalt(III) complexes: (Figure 1) K_3_[Co(CN)_6_] (**1**); [Co(NH_3_)_6_]Cl_3_ (**2**); [Co(en)_3_]Cl_3_ (**3**), en = ethylenediamine); [Co(tn)_3_]Cl_3_ (**4**), tn = trimethylenediamine); [Co(tame)_2_]Cl_3_ (**5**), tame = triaminomethylethane); and [Co(dinosar)]Cl_3_ (**6**), dinosar = dinitrosarcophagine). This series enables comparison of the temperature-dependent relaxation dynamics of these complexes with (*i*) molecular symmetry (e.g., from the *O*_h_ complexes **1** and **2** to the nearly *D*_3_ complexes **3–6**), and (*ii*) relative degree of encapsulation (from **2–6**). We further computed quadrupolar coupling parameters from computational structures to rationalize the relative temperature dependence of the relaxation dynamics. We find no precise correlation between relaxation and encapsulation. Instead, we propose that Δ*T*_1_/Δ*T* of the ^59^Co nucleus is driven by changes in the quadrupolar coupling parameters, Δ*e*^2^*qQ*, from thermally driven structures. These evaluations highlight important structural conditions of chelation among the series, which are shown to yield various trends in temperature-dependent *T*_1_, *T*_2_, and *T*_2_*.

## 2. Materials and Methods

### 2.1. General Considerations

Compounds utilized in this study were either purchased from commercial chemical vendors and used as received (**1** and **2**) or synthesized according previously reported literature preparations (**3**–**6**) [20–24].

### 2.2. Variable-Temperature ^59^Co-NMR Spectroscopy

Samples of all measured compounds were made as 0.7 mL volumes of 30 mM concentrations in protiated distilled water. Spectroscopic measurements were made at 118 MHz (^59^Co) using an Agilent Unity INOVA 500 MHz (^1^H) spectrometer at a field strength of 11.74 T with a 5mm BB NMR probe. Before any data collection, standard shims, deuterium locking, and probe tuning were made on 1 M sample of K_3_[Co(CN)_6_] in D_2_O, the ^59^Co-NMR reference standard. During ^59^Co-NMR experiments, data were collected in the absence of shimming and locking due to field stability of the instrument. Each sample was measured across a temperature range of 10–60 °C in 10 °C intervals. For each regulated temperature interval, samples were allowed to thermally equilibrate for 15 min before the probe was tuned for each pulse experiment.

### 2.3. Variable-Temperature ^59^Co Inversion Recovery and CPMG Experiments

Inversion recovery experiments were made on each sample across a temperature range of 10–60 °C in 10 °C intervals upon thermal equilibration. Inversion recovery data were acquired from 180°− τ − 90° pulse sequence experiments with 180° and 90° pulse lengths set at 22.4 and 11.2 μs, respectively. Pulse delay lengths τ were set by exponentially incremented time intervals relative to previously reported room temperature *T*_1_ values of each compound [19]. Similarly, CPMG (Carr–Purcell–Meiboom–Gill) pulse sequence experiments were made on each sample across a temperature range of 10–60 °C in 10 °C increments [25,26]. CPMG data were acquired from 90° − (τ − 180° − τ)_*n*_ spin echo pulse sequence experiments with 180° and 90° pulse lengths identical to the corresponding inversion recovery parameters.

### 2.4. Computation of ^59^Co Quadrupolar Coupling Constants

Computational analyses were completed for the Co–N_6_ encapsulation series (**2**–**6**) by structural optimizations over a range of temperatures. Temperature-specific optimizations were assisted by previous extended X-ray absorption fine-structure (EXAFS) characterization by fixing Co–N distances according to experimentally determined metal–ligand bond lengths to the three temperatures utilized in the EXAFS study, i.e., 13, 35, and 57 °C [27]. The remainder of the structure was allowed to optimize freely about the fixed Co–N_6_ coordination sphere using the Gaussian 16 [28] electronic structure package. Electronic properties calculations were then performed using Orca 4.11 [29] to predict the quadrupolar coupling constant parameter (*e*^2^*qQ*) of the temperature-specific optimized structures.

## 3. Results

**Figure 2.**
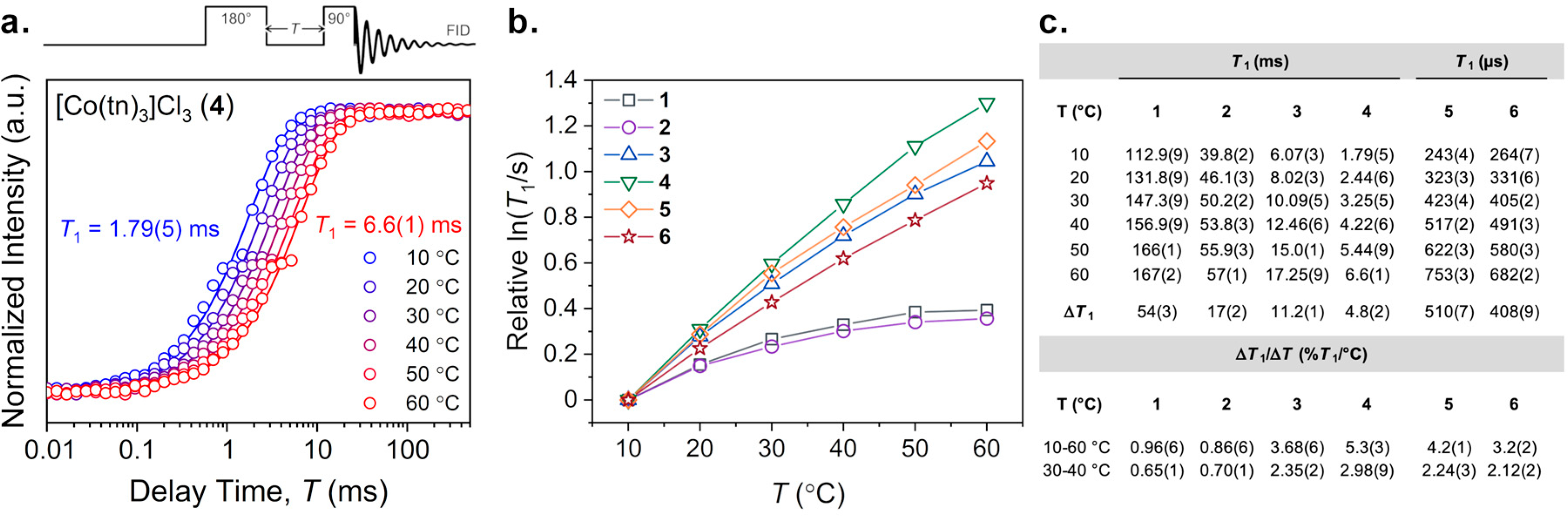
(**a**) Experimental variable-temperature (10–60 °C) inversion recovery measurements (circles) with exponential recovery fits (traces) for [Co(tn)_3_]Cl_3_ (**4**) on logarithmic scale. Temperature-specific *T*_1_ values were extracted from exponential decay fits. The general pulse sequence for the inversion recovery experiment is depicted. (**b**) Variable-temperature *T*_1_ plots of **1**–**6** on logarithmic scale showing relative changes. Error bars are within the width of the data points. Traces are guides for the eye. (**c**) Temperature-specific *T*_1_ spin–lattice relaxation times with error for **1**–**6** from 10–60 °C with absolute values of Δ*T*_1_ and relative values of Δ*T*_1_/Δ*T* temperature sensitivities.

The first temperature-dependent ^59^Co nuclear spin property we investigated was the spin–lattice, or *T*_1_, relaxation time. Variable-temperature inversion recovery experiments were performed for **1**–**6** over a 10–60 °C temperature range. At each temperature, an initially inverted ^59^Co-NMR peak was observed and intensity was recovered as a function of increasing delay time following the inverting π pulse. Figure 2a shows the resulting recovery curves of **4** obtained from these pulsed experiments at different temperatures. Additional inversion recovery curves are available in the supplementary information (Figures S1–S6). The fitted inversion recovery data for **1**–**6** reveal lengthening of *T*_1_ with increasing temperature. The observed ranges of *T*_1_ span from 112.9(9) to 167(2) ms for **1**, 39.8(2) to 57(1) ms for **2**, 6.07(3) to 17.25(9) ms for **3**, 1.79(5) to 6.6(1) ms for **4**, 243(4) to 753(3) μs for **5**, and 264(7) to 682(2) μs for **6** (Figure 2c). The largest absolute change in *T*_1_ over this temperature range is exhibited by **1** (Δ*T*_1_ = 54(3) ms), while the smallest difference occurs for **6** (Δ*T*_1_ = 408(9) μs). Between the minimum and maximum values of **1** and **6**, absolute changes in Δ*T*_1_ for **2**–**5** are 17(1) ms, 11.2(1) ms, 4.8(2) ms, and 511(7) μs, respectively. The general magnitudes of these values are consistent with previous ^59^Co relaxation data on structurally similar cobalt systems [30–33].

For the purpose of comparison, it is useful to define *relative* changes in *T*_1_ for each complex since absolute differences Δ*T*_1_, as above, heavily weight molecules with long *T*_1_ times. As a result, the use of logarithmic scales of *T*_1_ with temperature are necessary to show a clear comparison of Δ*T*_1_ between **1**–**6** (Figure S7). In the following discussion, we express a comparative degree of change in *T*_1_ between 10 to 60 °C as a percentage difference divided by the 50 °C window. For example, the Δ*T*_1_ of **1** over 10 to 60 °C is approximately 54 ms. This value corresponds to a 48.2% increase in *T*_1_ from 112.9 ms (10 °C) over the 50 °C window, thus quantitated by 0.96(6)%*T*_1_/°C. Similarly, the other relative Δ*T*_1_/Δ*T* sensitivities are 0.86(6), 3.68(6), 5.3(3), 4.2(1), and 3.2(2)%*T*_1_/°C for **2**–**6**, respectively. Figure 2b depicts the relative magnitudes of these values for all complexes over the 10–60 °C temperature window on a logarithmic scale. Owing to the potential utility of relaxation in modern biomedical imaging techniques, we highlight the aforementioned values of Δ*T*_1_/Δ*T* within the biologically relevant domain of 30–40 °C at 0.65(1), 0.70(1), 2.35(2), 2.98(9), 2.24(3), and 2.12(2)%*T*_1_/°C for **1**–**6**, respectively (Figure 2c). These values follow the same general trend as with the 10–60 °C window, though the changes in magnitude differ slightly.

Notably, **4** shows the greatest change for both temperature windows, and **1** and **2** show the smallest relative increase in *T*_1_. However, the relation between *T*_1_ and *T* show varying degrees of temperature linearity across the series. *T*_1_ is expected to show a linear temperature dependence if the quadrupolar mechanism is operative. A high degree of linearity is shown by the *D*_3_-symmetric molecules of the series, **3**–**6**. For these complexes, quadrupolar relaxation is expected due to the interaction between the electric quadrupolar moment and the lower-symmetry electric field gradient at the ^59^Co nucleus (relative to *O*_h_ **1** and **2**). However, the non-linear relaxation behaviors of **1** and **2** suggest different operative relaxation processes of the central ^59^Co nucleus [30,34]. For these complexes, curvature in the plots of ln(*T*_1_/s) vs. *T* (°C) (Figure 2b) show a gradual decline with increasing temperature, indicative of another contributing relaxation mechanism. The spin–rotation relaxation mechanism is known to contribute to relaxation in similar *O*_h_^59^Co complexes, [30,31] thus is the likely origin of the non-linear temperature dependence in **1** and **2**.

**Figure 3.**
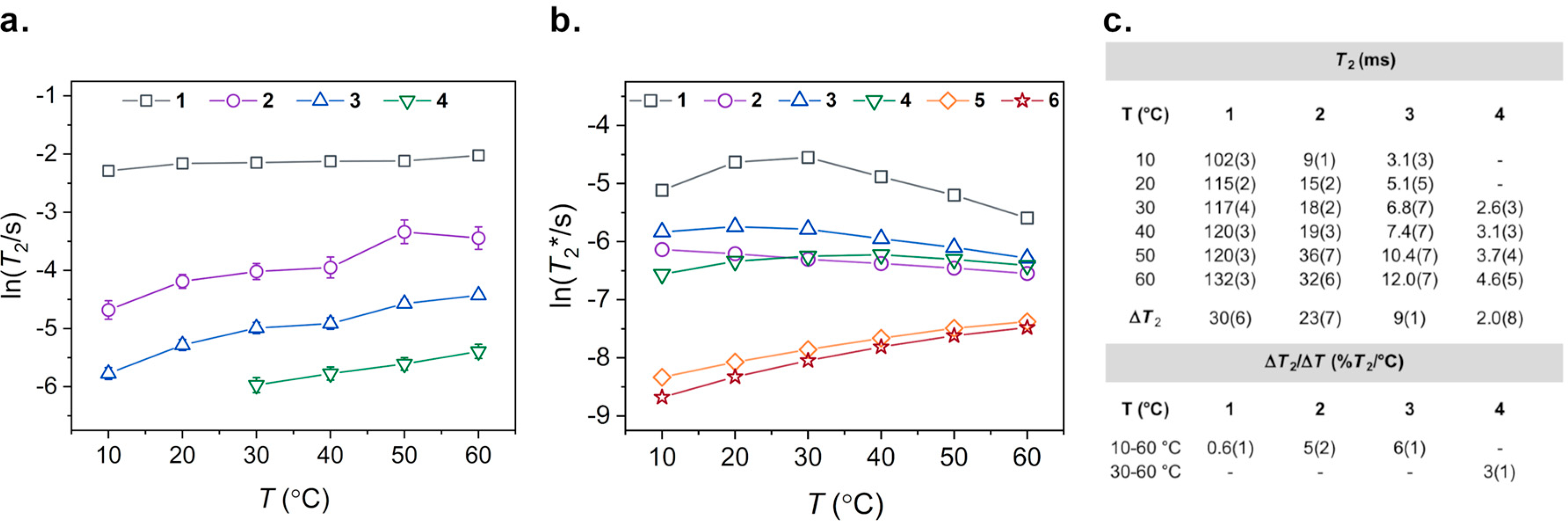
(**a**) Variable-temperature *T*_2_ plots of **1**–**4** on logarithmic scale showing relative changes in *T*_2_ spin–spin relaxation times. Error bars for K_3_[Co(CN)_6_] (**1**) are within the width of the data points. Traces in both plots are mean to guide the eye. (**b**) Variable-temperature *T*_2_* trends from linewidth analyses of **1**–**6** from 1D ^59^Co NMR spectra. (**c**) Temperature-specific *T*_2_ spin–spin relaxation times with error for **1**–**4** with absolute values of Δ*T*_2_ and relative values of Δ*T*_2_/Δ*T* temperature sensitivities.

The second temperature-dependent nuclear spin property we investigated was *T*_2_. Variable-temperature CPMG experiments were performed over a 10–60 °C temperature range for on **1**–**3**, and a 30–60 °C range for **4** to collect *T*_2_ values. At each temperature measurement, a ^59^Co NMR peak was observed with an intensity that decayed as a function of increasing number of π pulses. Figures S8–S11 show the resulting decay curves of the studied complexes and *T*_2_ times were determined from exponential fits of the decay. Similar to the temperature-dependent *T*_1_ behaviors, *T*_2_ increases with increasing temperature for **1**–**4**. Figure 3a shows the relaxation trends for **1**–**4** over the 50 °C window. Unfortunately, due to instrumental limitations, we were not able to collect *T*_2_ values for **4** at 10 and 20 °C, nor for **5** and **6** at any temperature between 10–60 °C. Pulse delay times for CPMG experiments on complexes with relatively low *T*_2_ values approached the same timescales as the pulse durations (on the order of 10–20 μs). Thus, CPMG data could not be collected for **5** and **6**, which are likely to have even shorter *T*_2_ times than **4** at 30 °C (the shortest experimentally determined *T*_2_ value). For **1**–**4**, the observed range of *T*_2_ times span from 102(3) to 132(3) ms for **1**, 9(1) to 32(6) ms for **2**, and 3.1(3) to 12.0(7) ms for **3** (Figure 3c). The largest absolute change in *T*_2_ over a 10–60 °C temperature range is exhibited by **1** (Δ*T*_2_ = 30(6) ms), followed by decreasing values of Δ*T*_2_ at 23(7) ms for **2**, and 9(1) ms for **3**. Between 30–60 °C, *T*_2_ for **4** was measured from 2.6(3) to 4.6(5) ms with an absolute Δ*T*_2_ of 2.0(8) ms. The increases in *T*_2_ over the studied range are expressed as Δ*T*_2_/Δ*T* by 0.6(1), 5(2), and 6(1)%*T*_2_/°C over 10–60 °C for **1**–**3**, respectively, while an increase of 3(1)%*T*_2_/°C is shown for **4** over 30–60 °C.

As an additional method of comparing the variation in ^59^Co nuclear spin properties of **1**–**6**, we investigated the dephasing time, or *T*_2_*, a relaxation time analogous to *T*_2_ above. *T*_2_* can be extracted from the temperature-dependent NMR linewidths through the relationship *T*_2_* = 1/(2πΔν) where Δν (Hz) is the full width at half the maximum height (FWHM) of the ^59^Co-NMR peak. This method enables a complete comparison of **1**–**6**, in contrast to the CPMG experiments. Figure 3b shows the temperature-dependent trends in *T*_2_* for all complexes over the 10–60 °C range. Complexes **1**, **3**, and **4** all show increasing *T*_2_* with increasing temperature up to a maximum, then begin to decrease with further increasing temperature. The maxima occur near 30, 20, and 40 °C for **1**, **3**, and **4**, respectively. In contrast, complex **2** shows a continual decline in *T*_2_* over the studied temperature range, while **5** and **6** both exhibit linear increases in *T*_2_*. The absolute changes in Δ*T*_2_* over 10–60 °C are −2.27, −0.73, −1.06, 0.24, 0.39, and 0.40 ms for **1**–**6**, respectively (Table S1). This trend is reflected in the smaller, biologically relevant 30–40 °C window, where absolute Δ*T*_2_* values are −2.99, −0.13, −0.46, 0.05, 0.08, and 0.08 ms. As with Δ*T*_1_ and Δ*T*_2_, the absolute difference in timescales heavily weights complexes with already long *T*_2_* values. The relative changes according to Δ*T*_2_*/Δ*T*, which here describe essentially the temperature dependence of the spectral linewidth, are −0.76, −0.67, −0.72, 0.33, 3.21, and 4.64%*T*_2_*/00B0C for **1**–**6**, respectively. The largest increase in *T*_2_* is shown by **6**, with **5** showing the second largest increase. This trend is reflected in the narrowing linewidths observed in the ^59^Co NMR spectra as a function of increasing temperature.

**Figure 4.**
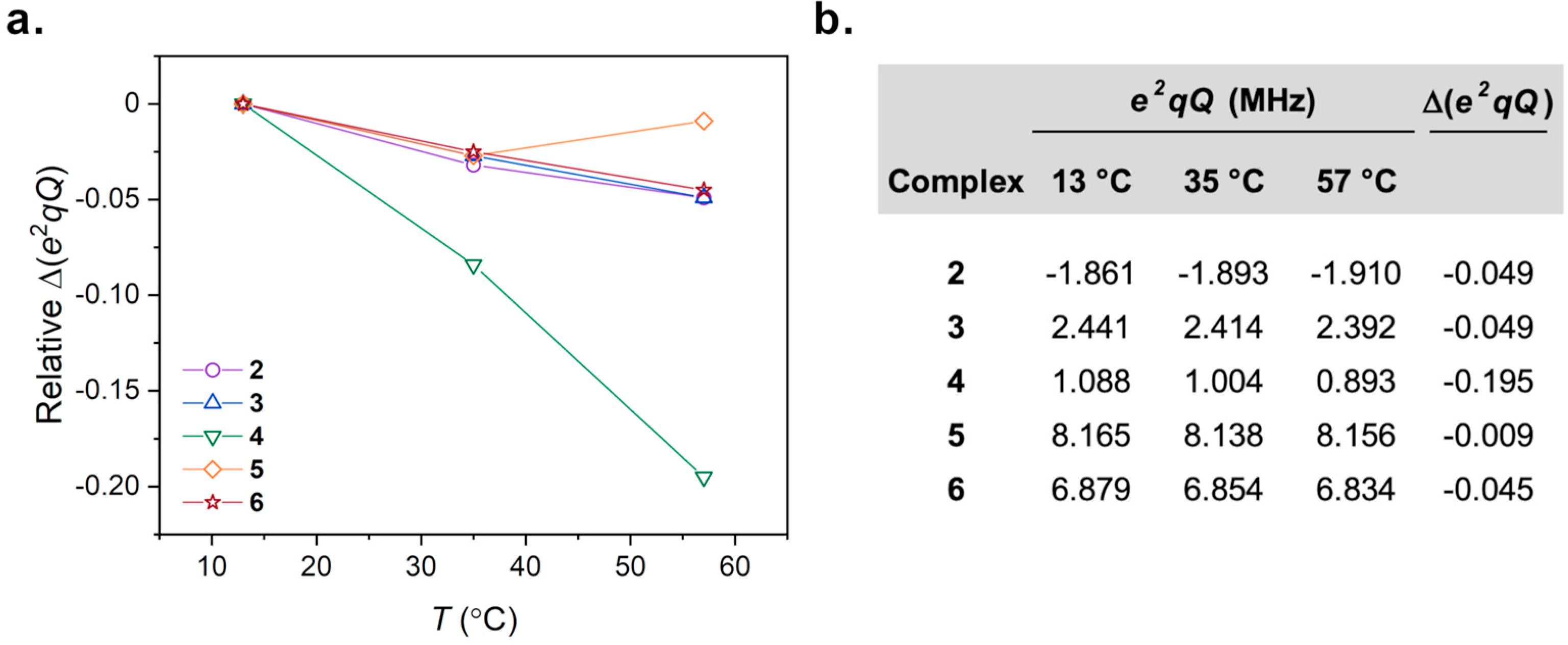
(**a**) Trends in predicted quadrupolar coupling parameters, *e*^2^*qQ,* from variable-temperature predicted structures of **2**–**6**. (**b**) Temperature-specific quadrupolar coupling parameters at each temperature-specific structure and Δ*e*^2^*qQ* over the ~50 °C range.

To assist in understanding the relaxation time data, we computed values of the quadrupolar coupling constant parameter (*e*^2^*qQ*) for the Co–N_6_ encapsulation series (**2**–**6**) at different temperatures within the 10–60 °C window. Predictions of *e*^2^*qQ* were completed from partially optimized, variable-temperature structures following analyses from extended X-ray absorption fine-structure (EXAFS) spectroscopy [27]. Values of *e*^2^*qQ* computed for these structures range from −1.861 to −1.910 MHz for **2**, 2.441 to 2.392 for **3**, 1.088 to 0.893 MHz for **4**, 8.165 to 8.156 MHz for **5**, and 6.879 to 6.834 MHz for **6** (Figure 4). The smallest values of *e*^2^*qQ* are found for the smaller complexes (**2**–**4**) reflecting higher symmetries in molecular structure, relative to the larger, more encapsulating *D*_3_ structures (**5** and **6**) showing the largest values of *e*^2^*qQ* in the series.

The differences in *e*^2^*qQ* by temperature-driven structure vary in scale, but all decrease with increasing temperature (Figure 4). Values of Δ*e*^2^*qQ* for **2**–**6** are found to be −0.049, −0.049, −0.195, −0.009, and −0.045 MHz, respectively. Of these predicted values, the greatest change is found for **4** followed by **2** and **3**, then **6** and **5**. Importantly, the largest Δ*e*^2^*qQ* is exhibited by **4** which also shows the largest Δ*T*_1_/Δ*T* value. Conversely, the encapsulated *D*_3_ structures of **5** and **6** possess the highest magnitudes of *e*^2^*qQ* between 8.156 to 8.165 MHz and 6.834 to 6.879 MHz, respectively, but show the least change by Δ*e*^2^*qQ*.

## 4. Discussion

**Figure 5.**
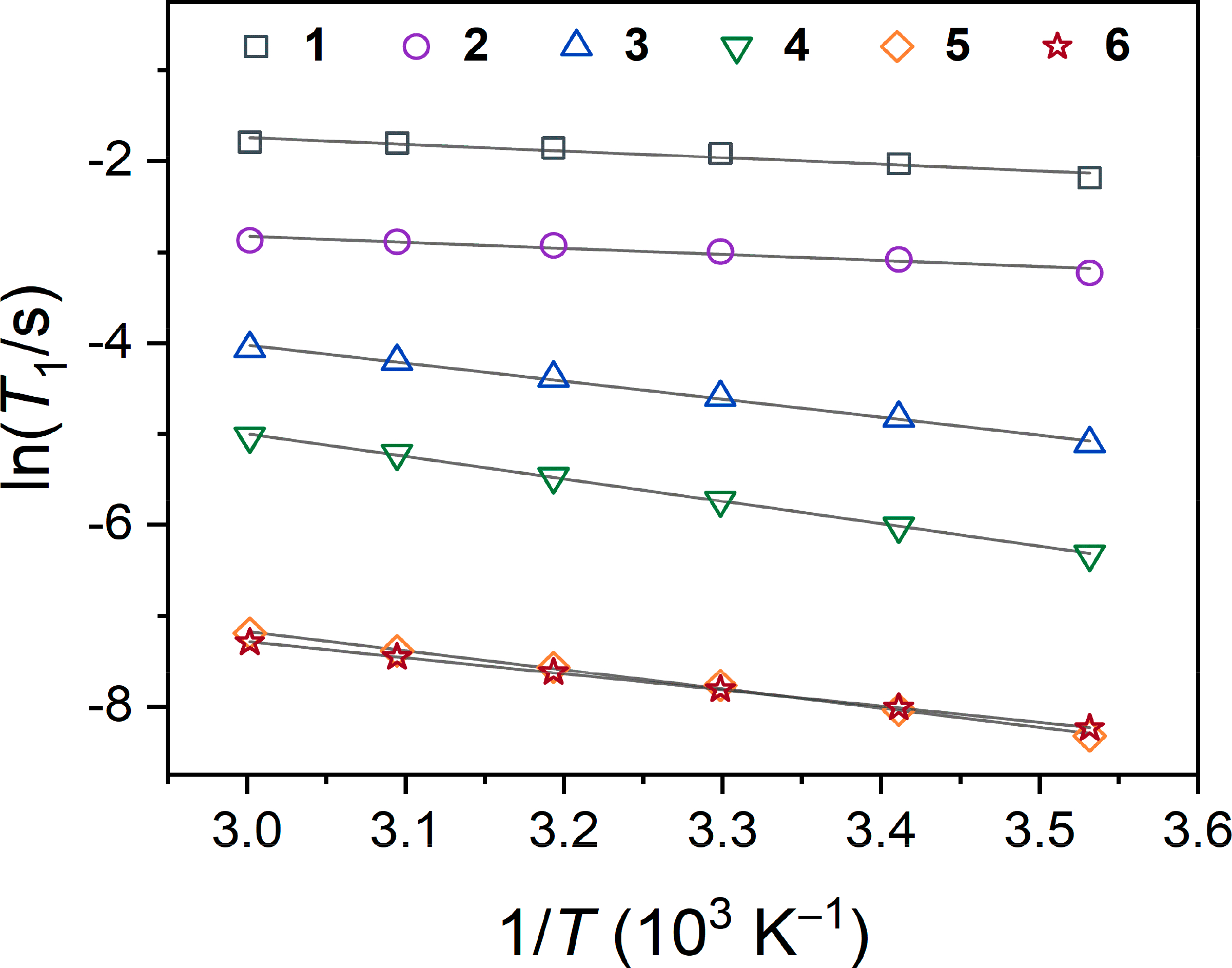
Arrhenius plots of variable-temperature *T*_1_ relaxation. Solid grey lines indicate linear regressions for **1–6**. Values of R^2^ from each fit (Table S2) are used to determine temperature linearity for each complex.

Spin–lattice relaxation of the ^59^Co nucleus is primarily attributed to the electric quadrupolar coupling interaction [30–32], which is dictated by the symmetry and structure of a given ligand shell. Evaluation of *T*_1_ via Arrhenius analyses of **1**–**6** elucidate the extent to which this is true. In principle, a higher linearity of ln(*T*_1_) vs. 1/*T* (10^3^ K^−1^) depicted in Figure 5 indicates the contribution of a single relaxation process in governing *T*_1_. A slightly curved temperature dependence is observed for *O*_h_ **1** and **2**, as evidenced by the lower R^2^ values (0.91) to linear regression. Conversely, highly linear trends are observed for the more *D*_3_-symmetry **3**–**6**, with R^2^ values of 0.99. For this latter series of four complexes, an activation energy, *E*_a_, can be extracted from these linear fits to the Arrhenius equation, 1/*T*_1_ = A exp(–*E*_a_/R*T*), where A is a preexponential factor, R is the ideal gas constant, and *T* is absolute temperature (Table S2). Here, *E*_a_ describes the activation energy to molecular tumbling, and a lower *E*_a_ suggests more facile motion in solution [30,35,36]. Activation energies for **3**–**6** are found to be 16.4(5), 20.6(3), 17.6(5), and 14.9(1) kJ/mol, respectively (1.37(4), 1.72(3), 1.47(4), and 1.24(1) × 10^3^ cm^−1^, respectively). Values of *E*_a_ increase from **6** < **3** < **5** < **4**, reflecting the same trend in Δ*T*_1_/Δ*T*. Notably, the moderately encapsulated complex **4** shows the highest barrier to rotation and also the highest Δ*T*_1_/Δ*T*. If the spin–lattice relaxation is expected to be driven by motional changes dependent on molecular mass, then the observed trend in Δ*T*_1_/Δ*T* cannot be strictly reasoned by changes in a temperature-dependent correlation time, τ_c_ (Figure S12 and Table S3). If the former were true, then the larger complexes **5** and **6** would be expected to have higher activation energies than that shown for **4**, an outcome that would be reflected by a longer τ_c_ in solution. In fact, they show shorter τ_c_ values, despite having larger ligand scaffolds. Thus, we conclude that the standard mechanisms for describing temperature-dependent relaxation, which principally stem from changes in correlation time, do not solely account for the observed changes here.

We instead propose that these changes in motion synergize with changes in the local symmetry of the ^59^Co nucleus to produce the observed trends in Δ*T*_1_/Δ*T*, especially in the series of *D*_3_ structures. Previous studies of **3**–**6** revealed ~0.007 Å changes in Co–N bond distances per °C over the 50 °C temperature range of our investigations here [27]. These changes in bond distances were also accompanied by changes in symmetry of the coordination geometry through changes in N–Co–N angles. As a result of these changes in symmetry, we find in our calculations here that the quadrupolar coupling constants decrease with increasing temperature with a magnitude that trends as **4** > **3** > **6** > **5** (Figure 4). The trend in Δ*e*^2^*qQ* does not completely correlate to the trend in relaxation across the series, hence our suggestion that motion is also important. However, complex **4** shows *both* the greatest value of Δ*e*^2^*qQ* at −0.194 MHz, and the highest Δ*T*_1_/Δ*T* at 5.3(3)%*T*_1_/Δ*T* over the 50 °C window.

The nearly equivalent values of *T*_1_ and *T*_2_ suggest that *T*_2_ is limited by *T*_1_, and, as such, *T*_2_ is also expected to be impacted by the quadrupolar coupling. However, the temperature dependence of *T*_2_ does not follow *T*_1_. Owing to the large temperature dependence of the ^59^Co chemical shift, we attribute this discrepancy to slight differences in resonance frequency by small temperature fluctuations which do not affect *T*_1_ as strongly as *T*_2_ [37]. We further highlight that the fast time scales of *T*_*2*_ for **5** and **6** are beyond the limits of the instrumentation. Hence, it would be challenging to utilize *T*_2_ as a thermometric parameter for these species. In that light, the temperature dependence of the ^59^Co linewidth appears more favorable for thermometry in complexes of greater encapsulation (and thus most chemically stable) owing to the linearity of Δ*T*_2_*/Δ*T* in the tridentate and encapsulated species **5** and **6**. Finally, we note that the values of *T*_2_* obtained here are likely lower bounds for this parameter, as temperature inhomogeneities in the instrument cavity (by even a fraction of 1 °C) will broaden the signal independent of *T*_2_*.

The above analyses suggest three important points for the development of ^59^Co spin-based probes for quadrupolar-driven relaxation thermometry. Firstly, we note the importance of chelating or macrocyclic ligands, as **3**–**6** exhibited mostly quadrupolar relaxation, which is likely driven by the *D*_3_-directing nature of these ligands. Secondly, we see that enabling a higher Δ*T*_1_/Δ*T* is largely dependent on whether the species possesses a strong temperature dependence of the quadrupolar coupling constant, not necessarily the magnitude of constant itself. Complex **4** exemplifies this point. Finally, third, the range of computed *e*^2^*qQ* and Δ*e*^2^*qQ* imply a tunable quadrupolar coupling interaction through temperature-driven structures. It is worth noting that this is, to the best of our knowledge, the first argument for this effect in governing thermometry by relaxation. Moreover, in this context, the most-encapsulated structures, **5** and **6**, both show the lowest Δ*e*^2^*qQ* values, compared to the structures of **3** and **4** with lesser denticity. This effect may be rationalized by a hindered variation in the *symmetry* of the structure due to the relative interconnectivity of the individual N donor atoms. Indeed, EXAFS analyses suggest that **4** exhibits the greatest transition *towards O*_h_ symmetry with increasing temperature when **3**, **5**, and **6** all deviate toward *D*_3_ symmetry [27]. This subtle difference in temperature-dependent structure is likely an important point toward designing future ^59^Co NMR thermometers.

## 5. Conclusions

We report a collection of temperature-dependent relaxation dynamic studies on a series of progressively encapsulated cobalt(III) complexes. The foregoing temperature-dependent data underline the fact that structure plays a vital role in controlling relaxation thermometry for the ^59^Co nucleus, but the coarse design principle of “encapsulation” does not solely govern the temperature dependence of *T*_1_ nor *T*_2_*. Relaxation times are found to be largely determined by the quadrupolar coupling interaction for the *D*_3_ complexes and a combination of quadrupolar and spin–rotation mechanisms for the *O*_h_ species (**1** and **2**). The chelated complex **4** has the largest relative increase in *T*_1_ as a function of its decrease in quadrupolar coupling, as mediated by a temperature-driven structure. We also found that encapsulated Co–N_6_ species, demonstrated by **5** and **6**, are potentially promising thermometric structures by linear *T*_2_* temperature dependencies. These factors thus provide a foundation for future studies of tuning temperature-dependent nuclear spin relaxation processes in Co(III) complexes.

## Supplementary Material

supplementary information
