# Supplementary material for: Ligand Control of 59Co Nuclear Spin Relaxation Thermometry
Source: Magnetochemistry. Author manuscript; Available in PMC 2021 Jun 3. (PMC8174815; doi:10.3390/magnetochemistry6040058)
Supplement: supplementary information [file NIHMS1696056-supplement-supplementary_information.pdf]

Electronic Supplementary Information for:

Ligand Control of ^{59}Co Nuclear-Spin Relaxation Thermometry

Tyler M. Ozvat, Spencer H. Johnson, Anthony K. Rappé, Joseph M. Zadrozny
Department of Chemistry, Colorado State University, 1301 Center Ave., Fort Collins, CO, 80523-1872

Table of Contents

**General Considerations**		S03
**Relaxation Fits and Linewidth Analysis**		S03
**Structure and Correlation Time Predictions**		S03
**Table S1**	Variable temperature ^{59}Co T_2* values and linewidth fit values	S05
**Table S2**	Linear trend fit parameters for ln(T_1) vs 1/T (10^3 K^{-1}) of **1-6**	S05
**Table S3**	Calculated variable-temperature correlation times of **2-6**	S06
**Figure S1**	Variable temperature inversion recovery fits of **1**	S06
**Figure S2**	Variable temperature inversion recovery fits of **2**	S07
**Figure S3**	Variable temperature inversion recovery fits of **3**	S07
**Figure S4**	Variable temperature inversion recovery fits of **4**	S08
**Figure S5**	Variable temperature inversion recovery fits of **5**	S08
**Figure S6**	Variable temperature inversion recovery fits of **6**	S09
**Figure S7**	T_1 trend analysis ln(T_1) vs. T (°C) of **1-6**	S09
**Figure S8**	Variable temperature CPMG fits of **4** from 30-60 °C	S10
**Figure S9**	Variable temperature CPMG fits of **1** from 10-60 °C	S10
**Figure S10**	Variable temperature CPMG fits of **2** from 10-60 °C	S11
**Figure S11**	Variable temperature CPMG fits of **3** from 10-60 °C	S11
**Figure S12**	Variable temperature correlation times of **2-6**	S12
**Table S4**	Computed structure of **3** at 13 °C	S12
**Table S5**	Computed structure of **3** at 35 °C	S13
**Table S6**	Computed structure of **3** at 57 °C	S14
**Table S7**	Computed structure of **4** at 13 °C	S15
**Table S8**	Computed structure of **4** at 35 °C	S16
**Table S9**	Computed structure of **4** at 57 °C	S18
**Table S10**	Computed structure of **5** at 13 °C	S19
**Table S11**	Computed structure of **5** at 35 °C	S20
**Table S12**	Computed structure of **5** at 57 °C	S21
**Table S13**	Computed structure of **6** at 13 °C	S23
**Table S14**	Computed structure of **6** at 35 °C	S24
**Table S15**	Computed structure of **6** at 57 °C	S26
**References**		S28

General Considerations

Studied compounds in this manuscript were either purchased from commercial chemical vendors or synthesized according previously reported literature preparations. Those compounds purchased were potassium hexacyanocobaltate(III) ($K_3[Co(CN)_6]$, **1**) and hexamminecobalt(III) chloride ($[Co(NH_3)_6]Cl_3$, **2**) and used in sample preparations as received. Compounds of study **3–6** were synthesized and are reported in previous work [1–5]. Characterization of **1-6** is detailed previously by us [6] where NMR spectra were collected on an Agilent Unity INOVA 500 MHz (^{1}H) spectrometer. UV-Vis spectra were collected on aqueous solutions of with an Agilent 8453 UV-Visible spectrophotometer. IR spectra were collected on solid powders with a Bruker TENSOR II FTIR spectrometer. Combustion analyses were performed by Robertson Microlit Laboratories.

Relaxation Fits and Linewidth Analysis

Fitting of all inversion recovery and CPMG experimental relaxation data was completed in Origin. A three-parameter exponential function (Eq. 1) was used to fit both sets of relaxation arrays in order to extract pertinent T_1 and T_2 relaxation values.

$$y = Ae^{\left(\frac{-x}{t_1}\right)} + y_0 \qquad \text{Eq. 1}$$

Where t_1 was solved for and taken directly as the experimental spin-lattice relaxation time, T_1 (s) from inversion recovery relaxation arrays (Figures S1-S6), and spin-spin relaxation time, T_2 (s) from CPMG experiments (Figures S8-S11). Values of T_2* were extracted the inverse relation of T_2* to the full-width half-maximum (FWHM) of 1D ^{59}Co-NMR spectra (Eq. 2).

$$T_2^* = 1/(2\pi\Delta\upsilon) \qquad \text{Eq. 2}$$

Where Δν (Hz) is the width of the FWHM extracted using a custom script written in MATLAB based on the findpeaks() method found in MATLAB's Signal Processing Toolbox [. All peak locations and widths were verified graphically before being used to calculate T_2* [7]. The specific script utilized herein is available upon reasonable request.

Structure and Correlation Time Predictions

Optimized molecular structures were computed for **3-6** using Gaussian 16 electronic software package [8]. Each optimization utilized the ωB97XD [9,10] functional and 6-311+g* basis set [11]. For each complex, three separate optimizations were performed with fixed metal-ligand bond lengths corresponding to previously determined experimental temperature-specific structures at 13, 35, and 57 °C by extended X-ray absorption fine structure (EXAFS). While the Co–N primary coordination sphere was restricted to experimental values, the reminder of the ligand structure was allowed to

minimize freely. The electronic properties of these experimentally-assisted predictions were then performed using ORCA 4.11 electronic software package [12] to determine values of the electric quadrupolar coupling constant (e^2qQ). Values of e^2qQ for each temperature-specific structure are compiled in Figure 4b on manuscript.

$$\frac{1}{T_1^Q} = \frac{3(2I+3)}{400I^2(2I-1)}\left(\frac{e^2Qq}{\hbar}\right)^2\left(1+\frac{\eta^2}{3}\right)\left(\frac{2}{1+\omega_x^2\tau_c^2}+\frac{8}{1+4\omega_x^2\tau_c^2}\right)\tau_c \qquad \text{Eq. 3}$$

In addition to e^2qQ, the asymmetry parameter, η was predicted from each temperature-specific structure. Together, these values were used to determine correlation times, τ_c from the canonical equation for T_1 (Eq. 3) [13,14]. The asymmetry parameters and quadrupole coupling constant vary for each complex and temperature. From these computational values, an equation of a single distinct coefficient was generated for each compound. The coefficients were plotted against the experimental temperatures and fit using a second-degree polynomial under the assumption that the coefficients varied continuously across the temperature range of 10-60°C. Each compound thus has a second degree polynomial as a function of temperature which outputs a single coefficient which is then used to generate coefficients for each temperature (10, 20, 30, 40, 50, and 60°C). Finally, the equation of T_1 as a function of rotational correlation time was solved by inserting the experimentally derived T_1 spin-lattice relaxation times and solving for τ_c. This process was accomplished via custom MATLAB script with the assistance of the curve-fitting and symbolic mathematics toolboxes [15,16]. All code used herein is available upon reasonable request.

Table S1. Temperature-specific T_2* dephasing times for **1-6** from 10-60 °C. Values were determined from full-width half-max (FWHM) linewidth analysis of 1D ^{59}Co NMR spectra.

	T_2* (ms)				T_2* (μs)	
**T (°C)**	**1**	**2**	**3**	**4**	**5**	**6**
10	6.00	2.16	2.93	1.41	240	170
20	9.76	2.02	3.21	1.77	312	242
30	10.57	1.83	3.07	1.93	387	320
40	7.58	1.70	2.61	1.98	471	405
50	5.53	1.57	2.25	1.83	560	492
60	3.73	1.43	1.87	1.65	626	566

Table S2. Arrhenius analysis of **1**-**6**. Linearity is determined from evaluations of R^2 values with error in slope and intercept values. Activation energy, E_a (kJ/mol) is calculated from the slope of ln(T_1) vs 1/T (10^3 K^{-1}) plots (see manuscript Figure 5).

	**1**	**2**	**3**	**4**	**5**	**6**
**R^2**	0.9138	0.9131	0.9953	0.9988	0.9964	0.9997
**Slope**	-0.7(1)	-0.66(9)	-1.98(6)	-2.4(4)	-2.12(6)	-1.79(1)
**Intercept**	2.3(2)	2.0(3)	6.0(2)	7.5(1)	6.4(2)	5.37(5)
**E_a (kJ/mol)**	6.2(8)	5.5(8)	16.4(5)	20.6(3)	17.6(5)	14.9(1)

Table S3. Calculated correlation times of **2-6** from 10-60 °C. Values of correlation times, τ_c are determined using Eq. 3 detailed above.

	τ_c (10^{-12} s)				
**T (°C)**	**2**	**3**	**4**	**5**	**6**
10	17.35	68.37	1150	153.25	190.31
20	14.63	52.34	891.31	115.58	152.26
30	13.19	42.03	722.32	88.69	124.83
40	12.15	34.35	610.67	72.49	103.29
50	11.57	28.87	526.34	60.2	87.53
60	11.32	25.21	488.02	49.47	74.61

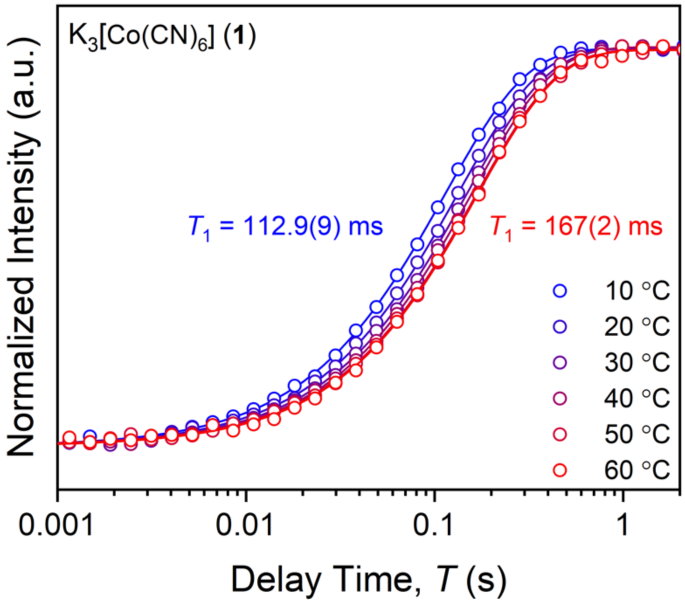

Figure S1. Variable temperature inversion recovery of complex **1** over a 10-60 °C temperature range at 30 mM concentration. Inversion recovery data (circles) are fit to relaxation curves (lines) to parameterize T_1 over a range of 112.9(9) to 167(2) ms with temperature.

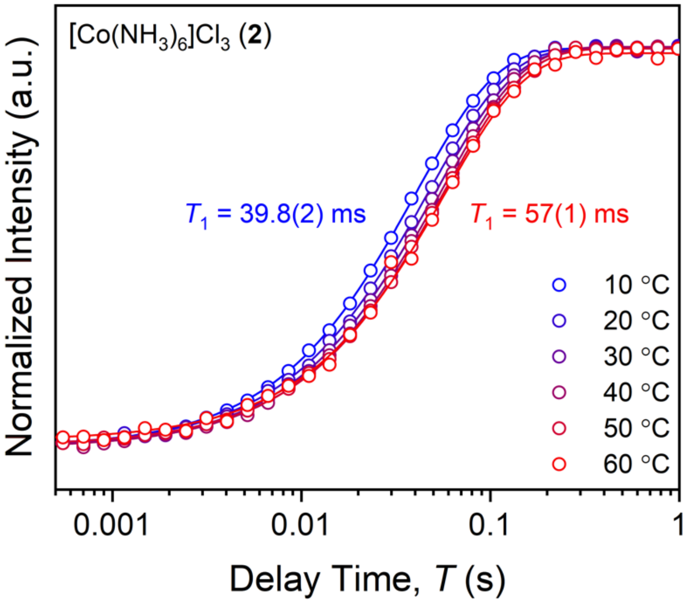

Figure S2. Variable temperature inversion recovery of complex **2** over a 10-60 °C temperature range at 30 mM concentration. Inversion recovery data (circles) are fit to relaxation curves (lines) to parameterize T_1 over a range of 39.8(2) to 57(1) ms with temperature.

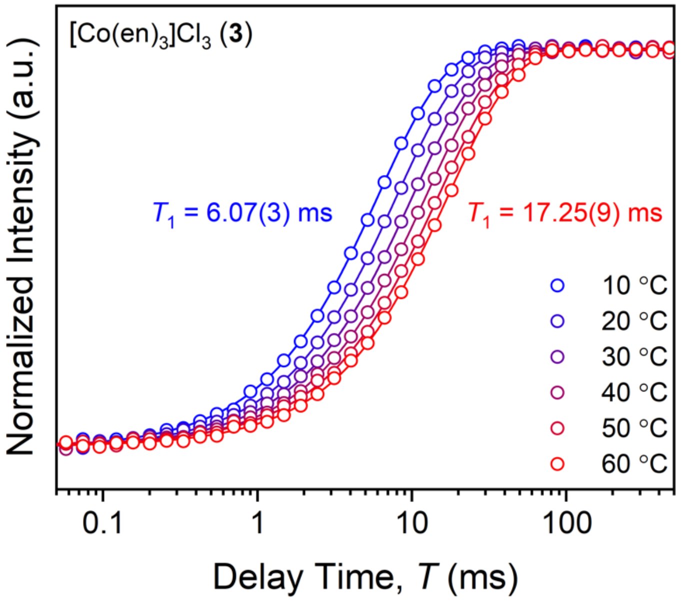

Figure S3. Variable temperature inversion recovery of complex **3** over a 10-60 °C temperature range at 30 mM concentration. Inversion recovery data (circles) are fit to relaxation curves (lines) to parameterize T_1 over a range of 6.07(3) to 17.25(9) ms with temperature.

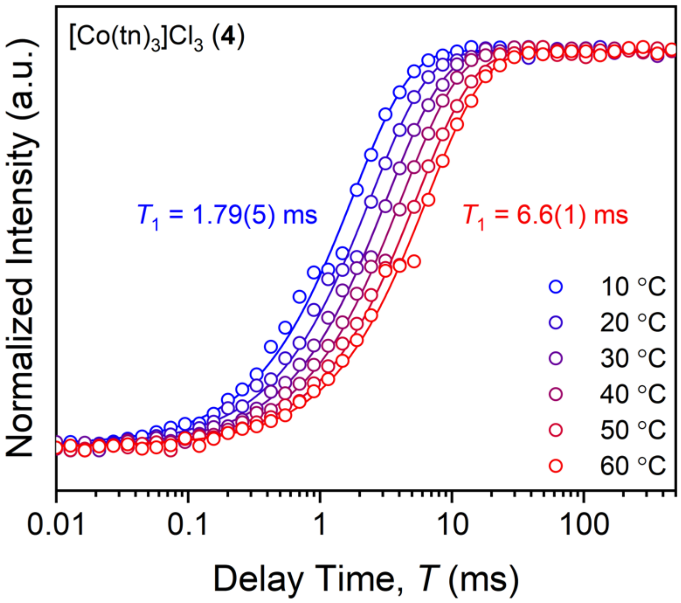

Figure S4. Variable temperature inversion recovery of complex **4** over a 10-60 °C temperature range at 30 mM concentration. Inversion recovery data (circles) are fit to relaxation curves (lines) to parameterize T_1 over a range of 1.79(5) to 6.6(1) ms with temperature.

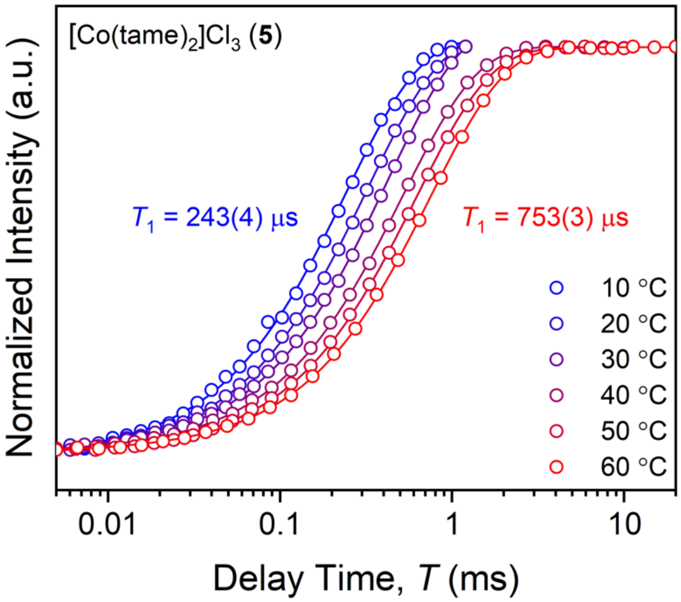

Figure S5. Variable temperature inversion recovery of complex **5** over a 10-60 °C temperature range at 30 mM concentration. Inversion recovery data (circles) are fit to relaxation curves (lines) to parameterize T_1 over a range of 243(4) to 753(3) μs with temperature.

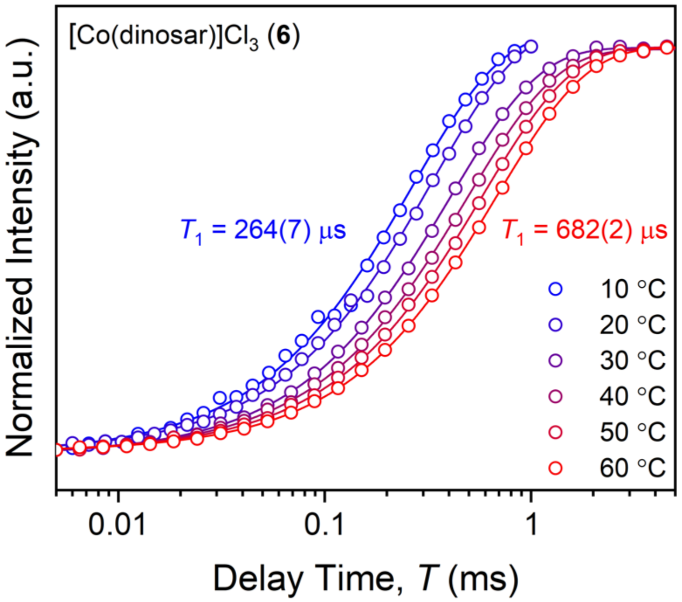

Figure S6. Variable temperature inversion recovery of complex **6** over a 10-60 °C temperature range at 30 mM concentration. Inversion recovery data (circles) are fit to relaxation curves (lines) to parameterize T_1 over a range of 264(7) to 682(2) μs with temperature.

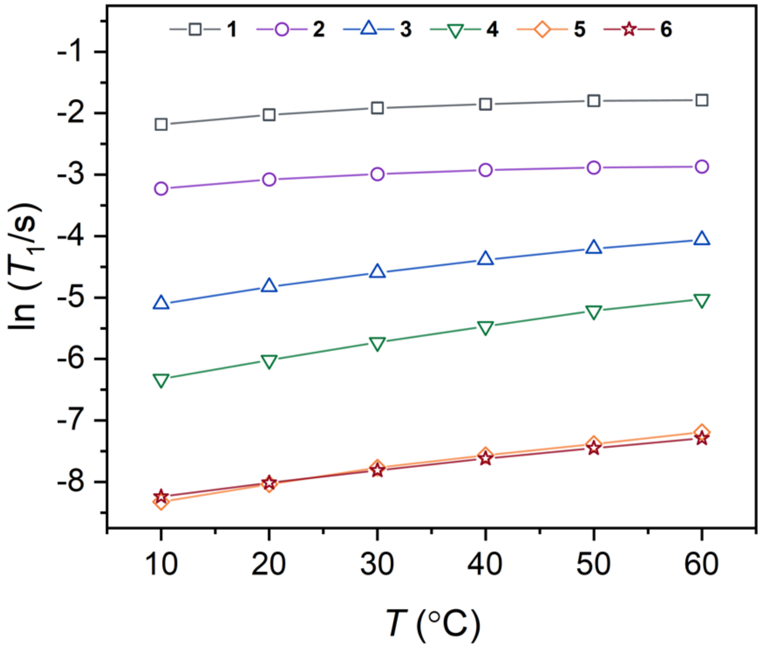

Figure S7. Variable temperature trends of ln(T_1/s) vs. T (°C) from fitted T_1 values. Colored traces are guides for the eye.

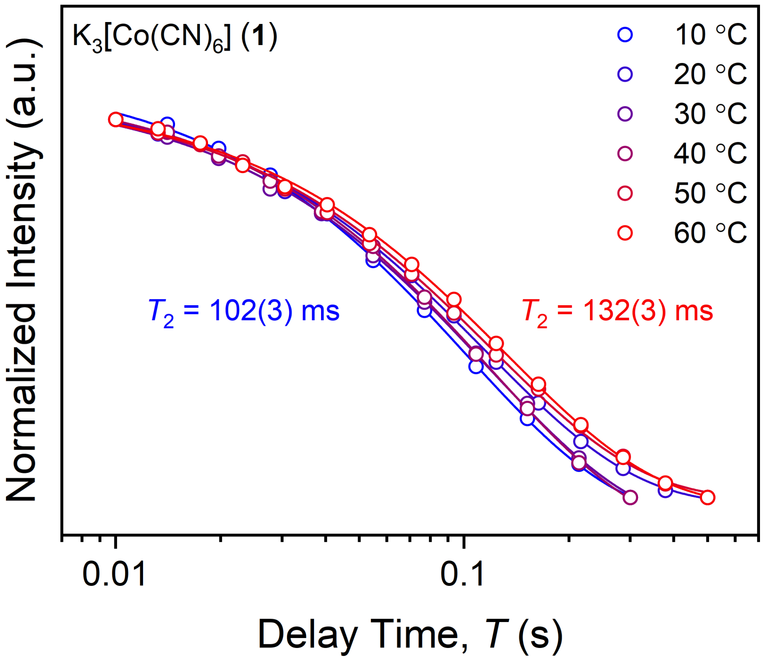

Figure S8. Variable temperature CPMG data of complex **1** over a 10-60 °C temperature range at 30 mM concentration. CPMG data (circles) are fit to relaxation curves (lines) to parameterize T_2 over a range of 102(3) to 132(3) ms with temperature.

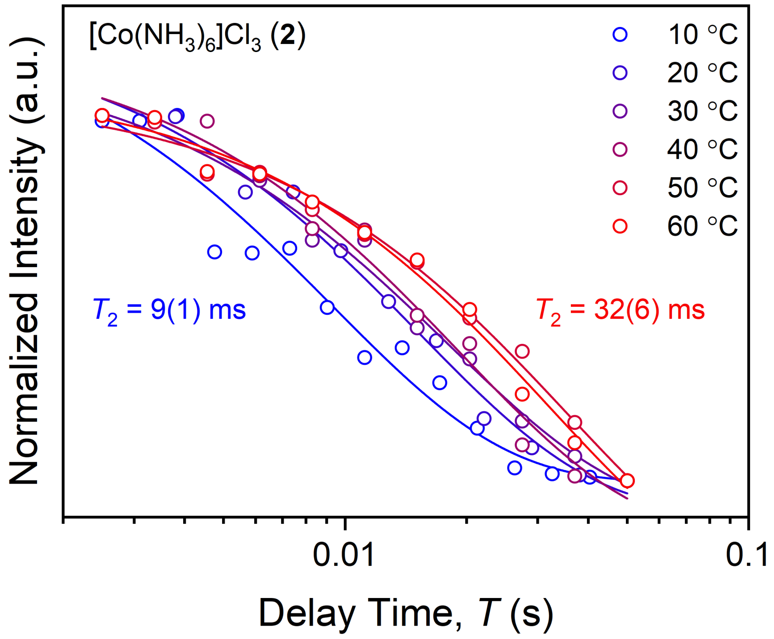

Figure S9. Variable temperature CPMG data of complex **2** over a 10-60 °C temperature range at 30 mM concentration. CPMG data (circles) are fit to relaxation curves (lines) to parameterize T_2 over a range of 9(1) to 32(6) ms with temperature.

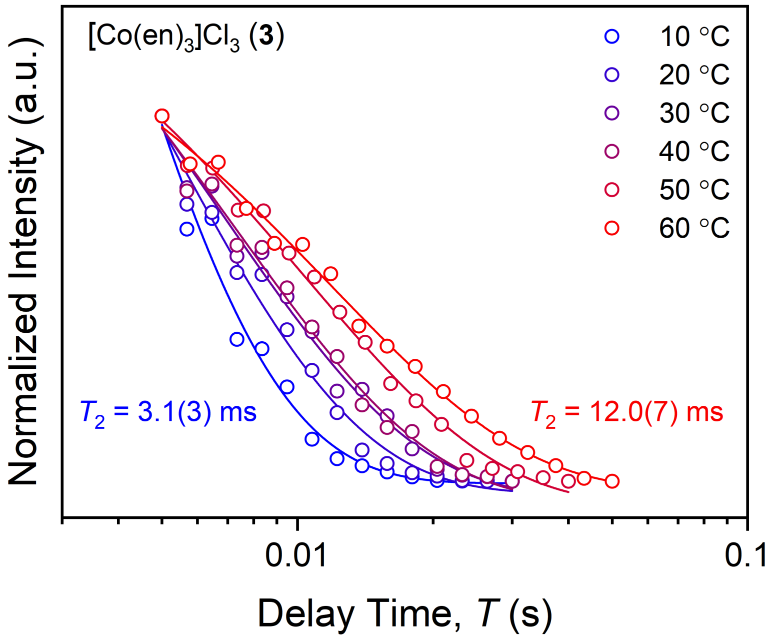

Figure S10. Variable temperature CPMG data of complex **3** over a 10-60 °C temperature range at 30 mM concentration. CPMG data (circles) are fit to relaxation curves (lines) to parameterize T_2 over a range of 3.1(3) to 12.0(7) ms with temperature.

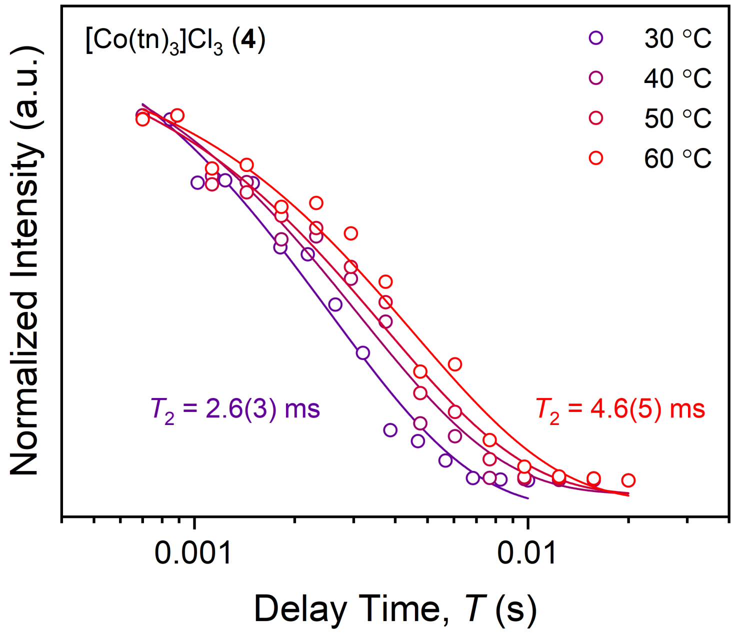

Figure S11. Variable temperature CPMG data of complex **4** over a 30-60 °C temperature range at 30 mM concentration. CPMG data (circles) are fit to relaxation curves (lines) to parameterize T_2 over a range of 2.6(3) to 4.6(5) ms with temperature.

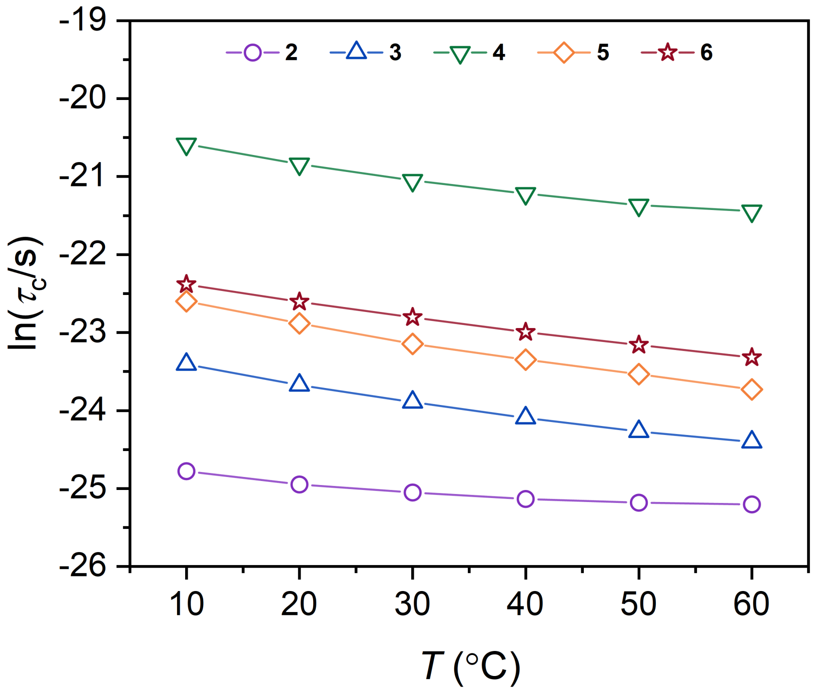

Figure S12. Variable-temperature correlation time, τ_c (s) trends on a logarithmic scale. Traces are guides for the eye. Calculated from values of τ_c are compiled in Table S3, determined from Eq. 3 (above).

Table S4. Computed coordinates of $[Co(en)_3]Cl_3$ (**3**) with fixed Co–N_6 bond distances from previous 13 °C EXAFS data (1.9694(5) Å). Total energy: -1953.43347520 Hartrees

Symbol	x	y	z
Co	0.00007964	-0.00016764	0.00031164
C	0.89173474	2.61316490	0.74934824
C	1.93710856	-1.96657225	-0.75055951
C	1.81808218	-2.07805438	0.74955723
H	2.33267589	0.03607462	-1.00890472
H	1.34069901	-0.54747408	-2.11808332
H	-0.20836619	-2.32363484	1.00909271
H	0.44794361	-1.37804768	2.11816861
H	-0.01304352	2.95425925	1.25776881
H	1.71480354	3.23941669	1.09739793
H	1.26909287	-2.66591634	-1.25866368
H	2.94996663	-2.17393474	-1.09966290
H	1.94948155	-3.10385570	1.09776376
H	2.56565918	-1.46460195	1.25776887
H	0.96971114	1.07658492	2.11793816

H	2.11693722	0.98039701	1.00921898
N	1.12140551	1.17432481	1.11460698
N	1.51985922	-0.57045710	-1.11481094
N	0.45704447	-1.55820877	1.11482917
N	-0.26612128	1.60160367	-1.11410483
N	-1.57760981	0.38357793	1.11480421
N	-1.25450187	-1.03166537	-1.11346394
C	0.73478814	2.66094359	-0.75065921
H	-0.19787894	1.43513017	-2.11752539
H	-1.19758324	2.00245317	-1.00684595
C	-2.70964608	-0.53377578	0.74943529
H	-1.90632569	1.34313450	1.00988501
H	-1.41701885	0.30042019	2.11807296
C	-2.67226774	-0.69412936	-0.75040950
H	-1.13604536	-2.03871317	-1.00570662
H	-1.14472574	-0.88993256	-2.11700203
H	1.67416681	2.43263471	-1.25948063
H	0.40747357	3.64181683	-1.09929780
H	-2.55401465	-1.48777875	1.25846218
H	-3.66332902	-0.13301734	1.09679191
H	-3.35815306	-1.46795921	-1.09904477
H	-2.94360960	0.23338406	-1.25982596

Table S5. Computed coordinates of $[Co(en)_3]Cl_3$ (**3**) with fixed Co–N_6 bond distances from previous 35 °C EXAFS data (1.9706(5) Å). Total energy: -1953.43368251 Hartrees

Symbol	x	y	z
Co	0.00000706	-0.00017152	0.00025222
C	0.17550713	2.75683253	0.74935382
C	2.38563166	-1.39131003	-0.75053436
C	2.29979109	-1.53002972	0.74974107
H	2.24330749	0.64527030	-1.00868895
H	1.43962576	-0.17722466	-2.11855791
H	0.40858394	-2.29819727	1.01004048
H	0.79447600	-1.21395733	2.11887920
H	-0.78716014	2.84885788	1.25759261
H	0.80590603	3.57659474	1.09772468
H	1.92416563	-2.24122598	-1.25861146
H	3.41739483	-1.32578378	-1.09974342
H	2.69488947	-2.48558464	1.09842992
H	2.86051377	-0.74195370	1.25768552

H	0.65418695	1.29511874	2.11840133
H	1.78605569	1.50277484	1.00940050
N	0.77442288	1.42856948	1.11481230
N	1.61749119	-0.15289548	-1.11495421
N	0.84997475	-1.38489635	1.11531141
N	-0.67647665	1.47713777	-1.11473294
N	-1.62412082	-0.04348867	1.11541907
N	-0.94132988	-1.32474338	-1.11449397
C	0.01216318	2.76161311	-0.75089377
H	-0.56689621	1.33505808	-2.11836161
H	-1.68054904	1.62028078	-1.00819176
C	-2.47512458	-1.22617111	0.75002811
H	-2.19400901	0.79568171	1.01036858
H	-1.44811812	-0.08114875	2.11893271
C	-2.39798108	-1.36996842	-0.75022530
H	-0.56360930	-2.26599225	-1.00767669
H	-0.87319502	-1.15919385	-2.11818921
H	0.97882745	2.78674451	-1.25923512
H	-0.56051345	3.62231871	-1.10018432
H	-2.07324124	-2.10590020	1.25805086
H	-3.50017578	-1.09014548	1.09864615
H	-2.85742396	-2.29612616	-1.09938277
H	-2.90288705	-0.54521676	-1.25848813

Table S6. Computed coordinates of $[Co(en)_3]Cl_3$ (**3**) with fixed Co–N_6 bond distances from previous 57 °C EXAFS data (1.9714(5) Å). Total energy: -1953.43280799 Hartrees

Symbol	x	y	z
Co	0.00019508	0.00004906	0.00060861
C	2.75615827	0.19294588	0.74938480
C	-1.06182947	-2.54904138	-0.75088589
C	-1.21193373	-2.48274823	0.74925518
H	0.93861878	-2.13851295	-1.00506315
H	0.01928172	-1.45134510	-2.11780278
H	-2.22332871	-0.70966434	1.00992409
H	-1.09728881	-0.94900061	2.11899024
H	2.71984672	1.15957439	1.25716555
H	3.65296814	-0.32234108	1.09726475
H	-1.96485164	-2.20381558	-1.25968768
H	-0.86092912	-3.56328343	-1.09985472
H	-2.10707252	-3.00122634	1.09666328

H	-0.35720846	-2.93496034	1.25762390
H	1.37091940	-0.47430937	2.11939392
H	1.72702692	-1.56974655	1.01095750
N	1.51975921	-0.57689452	1.11613569
N	0.06469696	-1.62455438	-1.11422003
N	-1.26005644	-1.02695564	1.11567238
N	1.37489372	0.86744891	-1.11481097
N	-0.25994628	1.60522255	1.11509697
N	-1.43824840	0.75621346	-1.11538221
C	2.73877837	0.35477460	-0.75085231
H	1.24780399	0.74021766	-2.11825263
H	1.38301300	1.88137356	-1.00699630
C	-1.54567948	2.28972792	0.74914383
H	0.49539222	2.28187817	1.00844047
H	-0.27296466	1.42592823	2.11854793
C	-1.67751974	2.19355820	-0.75105813
H	-2.32006227	0.25548143	-1.00845364
H	-1.26365264	0.71020521	-2.11868480
H	2.89146450	-0.60028826	-1.25885022
H	3.51666532	1.03568240	-1.10024273
H	-2.36384022	1.77411307	1.25732587
H	-1.54868110	3.32398979	1.09715355
H	-2.65649283	2.52592546	-1.10025295
H	-0.92743153	2.80413543	-1.25910970

Table S7. Computed coordinates of $[Co(tn)_3]Cl_3$ (**4**) with fixed Co–N_6 bond distances from previous 13 °C EXAFS data (1.9825(5) Å). Total energy: -2071.43715806 Hartrees

Symbol	x	y	z
Co	0.00082394	-0.00002603	-0.09780114
C	-1.67282784	-2.17837128	1.24936642
C	-1.77491496	-2.90315556	-0.07575167
C	-0.45650228	-2.95717548	-0.81567793
C	2.72373640	-0.35588755	1.25023271
C	3.40342413	-0.08269594	-0.07456158
C	2.78952128	1.08354880	-0.81730302
C	-1.05366242	2.53618474	1.24881154
C	-1.63271379	2.98669205	-0.07539859
C	-2.33446143	1.87051619	-0.81725600
H	-2.32720288	-0.32451903	0.73126376
H	0.41907415	1.33548225	1.99310469

H	-1.98804116	0.03629590	-1.61380668
H	-0.95854105	1.14261209	-2.12762713
H	-0.85379547	-2.58105273	1.84953281
H	-2.58662465	-2.30451453	1.83258381
H	-2.09174256	-3.92988912	0.11991995
H	-2.56551387	-2.47155450	-0.70155007
H	0.31912715	-3.38878179	-0.17824541
H	-0.52689688	-3.59751468	-1.69644582
H	2.66029410	0.55557102	1.84890315
H	-1.35844103	-0.30601111	1.99696177
H	3.29070317	-1.08209232	1.83525507
H	4.45033630	0.15869951	0.12176674
H	3.42733950	-0.98422107	-0.69877502
H	2.77380214	1.97243504	-0.18182372
H	3.37933387	1.34169038	-1.69833838
H	-1.80950426	2.02467818	1.84901788
H	-0.70867367	3.39119469	1.83278618
H	-2.36652370	3.77124442	0.12169140
H	-0.86559572	3.45953241	-0.70061152
H	-3.09585915	1.41190671	-0.18150223
H	0.96371313	-1.74425618	-1.61006990
H	-2.85369763	2.25109730	-1.69833702
H	-0.50697896	-1.40254490	-2.12753042
H	1.44978775	-1.85200810	0.72960481
H	0.94638533	-1.02549379	1.99625442
H	1.02889230	1.70249829	-1.61480012
H	1.47028817	0.25694854	-2.12763835
H	0.87830325	2.18373158	0.72399074
N	-1.44260765	-0.70684239	1.06281267
N	0.01216135	-1.59799250	-1.27103737
N	1.33570777	-0.89553023	1.06248588
N	1.37871252	0.80669814	-1.27284486
N	0.10896238	1.60574993	1.05973859
N	-1.38802044	0.78814600	-1.27253716

Table S8. Computed coordinates of $[Co(tn)_3]Cl_3$ (**4**) with fixed Co–N_6 bond distances from previous 35 °C EXAFS data (1.9881(5) Å). Total energy: -2071.43959515 Hartrees

Symbol	x	y	z
Co	-0.00047770	0.00001042	-0.09864853
C	1.65713891	2.19355690	1.25071602

C	1.75385978	2.91927955	-0.07445244
C	0.43591109	2.96392578	-0.81621854
C	-2.72902004	0.33678225	1.25098215
C	-3.40625076	0.05731522	-0.07392494
C	-2.78482696	-1.10512844	-0.81670927
C	1.07321152	-2.53103862	1.25032354
C	1.65492809	-2.97724353	-0.07429306
C	2.35008781	-1.85720121	-0.81687628
H	2.32936170	0.34598104	0.73108147
H	-0.40870314	-1.34150001	1.99578641
H	1.99210579	-0.02637329	-1.61780756
H	0.96964749	-1.14077050	-2.12993827
H	0.83344980	2.58814275	1.84990310
H	2.56899456	2.32917067	1.83490344
H	2.06242557	3.94842113	0.12179462
H	2.54843360	2.49421810	-0.69968032
H	-0.34406204	3.38821371	-0.17916260
H	0.50278324	3.60664848	-1.69555684
H	-2.65777072	-0.57387020	1.84999143
H	1.36138157	0.31739414	1.99816940
H	-3.30286161	1.05798436	1.83554743
H	-4.45133673	-0.19154465	0.12282760
H	-3.43704034	0.95823808	-0.69869428
H	-2.76093158	-1.99321373	-0.18037356
H	-3.37494251	-1.36864382	-1.69598996
H	1.82530377	-2.01304023	1.84968466
H	0.73625957	-3.38897494	1.83476756
H	2.39368341	-3.75715564	0.12274859
H	0.89079902	-3.45509497	-0.69936497
H	3.10711245	-1.39222022	-0.18055386
H	-0.97395352	1.74113529	-1.61519611
H	2.87354086	-2.23603489	-1.69624913
H	0.50081316	1.41056284	-2.13018196
H	-1.46708545	1.84374346	0.72970512
H	-0.95704180	1.02171084	1.99752871
H	-1.02098006	-1.71146317	-1.61827077
H	-1.47390793	-0.26836428	-2.12982665
H	-0.86243109	-2.19380974	0.72650076
N	1.44202919	0.72015104	1.06458556
N	-0.02172985	1.60257226	-1.27503540
N	-1.34611266	0.88867714	1.06414130

N	-1.37761739	-0.81858090	-1.27590666
N	-0.09714474	-1.61075224	1.06266165
N	1.39766321	-0.78224721	-1.27585590

Table S9. Computed coordinates of $[Co(tn)_3]Cl_3$ (**4**) with fixed Co–N_6 bond distances from previous 57 °C EXAFS data (1.9910(5) Å). Total energy: -2071.43833732 Hartrees

Symbol	x	y	z
Co	-0.00091913	0.00005460	-0.09993031
C	1.72968398	2.13716024	1.25236300
C	1.85144179	2.86009847	-0.07230998
C	0.53644922	2.94992895	-0.81524822
C	-2.71742800	0.42655991	1.25256303
C	-3.40500936	0.16936571	-0.07155763
C	-2.82267295	-1.01201418	-0.81618102
C	0.98951459	-2.56553382	1.25119378
C	1.55848757	-3.03101633	-0.07235868
C	2.28925096	-1.93460636	-0.81601186
H	2.34132165	0.26906883	0.73050887
H	-0.45779294	-1.33147320	1.99363145
H	1.98946628	-0.09600752	-1.62353816
H	0.93088217	-1.17886382	-2.13045624
H	0.91876822	2.55806185	1.85103449
H	2.64498254	2.24285247	1.83737078
H	2.19439061	3.87812225	0.12486583
H	2.63166053	2.40889669	-0.69730107
H	-0.22950832	3.39928041	-0.17841510
H	0.62603808	3.59136791	-1.69351736
H	-2.67443393	-0.48607554	1.85122464
H	1.37320310	0.27046558	1.99824246
H	-3.26757078	1.16536513	1.83795045
H	-4.45726013	-0.04589608	0.12672119
H	-3.40781805	1.07112552	-0.69585205
H	-2.82586075	-1.90074000	-0.18032877
H	-3.42307162	-1.25584902	-1.69418928
H	1.75661485	-2.07054049	1.85094231
H	0.62567150	-3.41220418	1.83595691
H	2.27228444	-3.83342082	0.12643404
H	0.78040694	-3.48546184	-0.69764691
H	3.05985762	-1.49237972	-0.17978890
H	-0.91269171	1.77637865	-1.61836573

H	2.80144699	-2.33157641	-1.69402653
H	0.55100365	1.39630437	-2.13101869
H	-1.40888765	1.89244371	0.72838000
H	-0.92373748	1.05619792	1.99704980
H	-1.08177060	-1.67342844	-1.62486358
H	-1.48821784	-0.21430680	-2.13037627
H	-0.93346219	-2.16888362	0.72203913
N	1.46708930	0.67173276	1.06530006
N	0.03373440	1.60560892	-1.27677211
N	-1.31810450	0.93461726	1.06436240
N	-1.40824592	-0.77055848	-1.27872162
N	-0.15177676	-1.61004144	1.06144599
N	1.37167413	-0.83135239	-1.27836670

Table S10. Computed coordinates of $[Co(tame)_2]Cl_3$ (**5**) with fixed Co–N_6 bond distances from previous 13 °C EXAFS data (1.9700(5) Å). Total energy: -2109.54797542 Hartrees

Symbol	x	y	z
Co	-0.00000347	0.00026855	0.00013800
N	-1.16688756	-1.36565803	0.80857250
N	-1.16696618	-0.01657170	-1.58695199
N	1.16676891	-1.43273672	-0.68256134
N	-1.16734922	1.38306928	0.77866627
N	1.16708267	1.30778504	-0.89944564
N	1.16729811	0.12585389	1.58209106
C	-4.64680352	-0.00048948	-0.00030824
C	-3.11426948	-0.00032950	-0.00007533
C	-2.60840971	-0.99667970	1.04747613
C	-2.60810793	-0.40917475	-1.38666869
C	-2.60868229	1.40509670	0.33918814
C	2.60826729	-1.08671343	-0.95383579
C	3.11426447	-0.00023486	-0.00017904
C	4.64681594	-0.00052409	-0.00019484
C	2.60847624	-0.28292936	1.41761373
C	2.60857697	1.36906715	-0.46396050
H	-1.17927408	-2.17178325	0.18471381
H	-0.80696977	-0.57782351	-2.35828537
H	0.80665405	-1.89602002	-1.51638867
H	-1.17997696	1.24590158	1.78872434
H	1.17923630	1.08134022	-1.89329024
H	0.80760127	-0.36408281	2.40072487

H	-5.03823579	-0.94835206	-0.37485339
H	-5.03853196	0.14911460	1.00771240
H	5.03838498	0.05747337	-1.01767911
H	-3.20353192	-1.91097901	1.02017900
H	-2.69895608	-1.48658235	-1.53877537
H	-2.70038243	2.07558247	-0.51769056
H	3.20329668	-1.99490842	-0.84440876
H	2.69952799	-1.34231745	1.66583596
H	3.20387882	1.72857178	-1.30491224
H	-0.80689062	-1.75209423	1.68070945
H	-1.17997355	0.92696462	-1.97264836
H	1.17920999	-2.17956753	0.01117579
H	-0.80782756	2.33182585	0.67758156
H	0.80731307	2.26165035	-0.88315728
H	1.18025773	1.10015339	1.88164753
H	-5.03831812	0.79772414	-0.63398939
H	5.03817798	-0.91082842	0.45821381
H	5.03854261	0.85151785	0.55884749
H	-2.70025774	-0.59009094	2.05666919
H	-3.20369694	0.07083907	-2.16494168
H	-3.20413793	1.83859331	1.14439121
H	2.70008237	-0.77266469	-1.99553305
H	3.20416550	0.26513040	2.14947802
H	2.70033074	2.11373663	0.32929369

Table S11. Computed coordinates of $[Co(tame)_2]Cl_3$ (**5**) with fixed Co–N_6 bond distances from previous 35 °C EXAFS data (1.9698(5) Å). Total energy: -2109.54887608 Hartrees

Symbol	x	y	z
Co	0.00000097	0.00072136	0.00018597
N	1.16625438	-1.22688610	-1.00626598
N	1.16706731	-0.25646809	1.56604750
N	-1.16624230	-1.51940555	0.45757358
N	1.16720556	1.48542682	-0.55963163
N	-1.16738390	1.15656640	1.08708403
N	-1.16692513	0.36488948	-1.54441032
C	4.64678658	-0.00140353	-0.00003446
C	3.11409782	-0.00066163	-0.00015564
C	2.60797535	-0.82642351	-1.18663075
C	2.60798268	-0.61506824	1.30837059
C	2.60890918	1.44005620	-0.12221780

C	-2.60776433	-1.21883315	0.77835707
C	-3.11410115	-0.00088924	-0.00010227
C	-4.64675639	-0.00149225	-0.00045140
C	-2.60790341	-0.06516864	-1.44429970
C	-2.60922217	1.28237159	0.66614310
H	1.17854770	-2.11848127	-0.51214917
H	0.80706155	-0.92708393	2.24452801
H	-0.80616597	-2.10404511	1.21135349
H	1.17970409	1.50323728	-1.57882998
H	-1.17949465	0.78235538	2.03525345
H	-0.80684594	0.00634557	-2.42840392
H	5.03775152	-0.99517605	0.22693691
H	5.03862902	0.29865023	-0.97387286
H	-5.03844289	-0.09854110	1.01398610
H	3.20256966	-1.73460406	-1.29832066
H	2.69820354	-1.70313731	1.29520854
H	2.70091450	1.97310264	0.82626181
H	-3.20254208	-2.10025785	0.53312317
H	-2.69827228	-1.07460599	-1.85058157
H	-3.20437046	1.50962570	1.55212015
H	0.80634568	-1.47660876	-1.92699805
H	1.18112734	0.61833775	2.08925675
H	-1.17878072	-2.15234719	-0.34146388
H	0.80831522	2.40808577	-0.31560748
H	-0.80865173	2.10236077	1.21524170
H	-1.18094868	1.37357461	-1.69148136
H	5.03857955	0.69164267	0.74703192
H	-5.03784634	-0.83178717	-0.59171792
H	-5.03856541	0.92541617	-0.42386462
H	2.69987786	-0.27151409	-2.12247784
H	3.20370959	-0.25895001	2.15045728
H	3.20413306	1.99038595	-0.85273026
H	-2.69920955	-1.06551568	1.85554883
H	-3.20355929	0.58743209	-2.08471047
H	-2.70165623	2.13883980	-0.00477191

Table S12. Computed coordinates of $[Co(tame)_2]Cl_3$ (**5**) with fixed Co–N_6 bond distances from previous 57 °C EXAFS data (1.9707(5) Å). Total energy: -2109.54796698 Hartrees

Symbol	x	y	z
Co	-0.00002601	0.00074305	0.00055857

N	-1.16688893	-1.54889778	0.34769887
N	-1.16733941	0.47520857	-1.51460612
N	1.16675876	-1.15213634	-1.09166143
N	-1.16810024	1.07590680	1.16807191
N	1.16767954	1.52238978	-0.45167702
N	1.16772835	-0.36794571	1.54454054
C	-4.64682460	-0.00154565	-0.00119689
C	-3.11412683	-0.00082791	-0.00051572
C	-2.60842817	-1.27169381	0.68852041
C	-2.60803758	0.03871999	-1.44557743
C	-2.60969110	1.23131967	0.75641952
C	2.60821662	-0.73969065	-1.24293472
C	3.11427754	-0.00076721	-0.00048792
C	4.64695022	-0.00148864	-0.00069595
C	2.60846886	-0.70707874	1.26107404
C	2.60951949	1.44510545	-0.01941544
H	-1.17864676	-2.12277741	-0.49468067
H	-0.80677893	0.18135110	-2.42188505
H	0.80647093	-1.33571692	-2.02762587
H	-1.18032488	0.63333990	2.08627004
H	1.17922811	1.61357971	-1.46689901
H	0.80765614	-1.08528670	2.17329448
H	-5.03765208	-0.78714590	-0.65084548
H	-5.03866420	-0.17150296	1.00355136
H	5.03852600	0.36689361	-0.95091189
H	-3.20353399	-2.13294357	0.38048083
H	-2.69793646	-0.93916962	-1.92286281
H	-2.70185214	2.13368231	0.14862430
H	3.20324055	-1.63726316	-1.41932402
H	2.69853199	-1.79138387	1.16989326
H	3.20474362	2.04584415	-0.70905859
H	-0.80705120	-2.18569275	1.05802044
H	-1.18119961	1.49184356	-1.58780778
H	1.17882710	-2.07632818	-0.66170068
H	-0.80936177	2.00981834	1.36466970
H	0.80878644	2.42471475	-0.14101085
H	1.18162678	0.46749007	2.12848500
H	-5.03858883	0.95345192	-0.35680223
H	5.03791828	-1.00886567	0.15516446
H	5.03882367	0.63688084	0.79356489
H	-2.70034793	-1.19588617	1.77386806

H	-3.20431575	0.73503032	-2.03755347
H	-3.20537625	1.39429482	1.65606938
H	2.69999732	-0.11896823	-2.13648700
H	3.20491356	-0.41235309	2.12607724
H	2.70222954	1.90901407	0.96463377

Table S13. Computed coordinates of [Co(dinosar)]Cl_3 (**6**) with fixed Co–N_6 bond distances from previous 13 °C EXAFS data (1.9701(5) Å). Total energy: -2672.10724547 Hartrees

Symbol	x	y	z
Co	-0.00001941	0.00000970	-0.00322799
N	4.58102675	-0.00318521	0.00587450
O	5.15176360	1.05038301	0.11873404
O	5.06236875	-1.10611100	-0.08226495
C	3.04251516	0.03605914	-0.00689277
C	2.61600956	-0.63741035	-1.30755835
C	2.60394321	1.49247291	0.06592851
C	2.61923168	-0.76362026	1.22367696
N	1.13357426	-0.85695034	-1.36773535
N	1.11224491	1.62460936	-0.07279474
N	1.13396349	-0.73572627	1.42997503
C	0.77475115	-2.30690051	-1.41543190
C	0.72523630	2.39193991	-1.29530838
C	0.75075218	-0.06252143	2.70786313
H	0.85800159	-0.49049625	-2.27538880
H	0.83910099	2.22031936	0.70475221
H	0.87764424	-1.71114226	1.56135256
N	-4.58106245	0.00335145	0.00593089
O	-5.15184120	-1.05032380	0.11758247
O	-5.06235369	1.10637325	-0.08114332
C	-3.04255293	-0.03595083	-0.00692876
C	-2.60405438	-1.49245668	0.06446588
C	-2.61608262	0.63872278	-1.30696591
C	-2.61921188	0.76260036	1.22436671
N	-1.11230278	-1.62452641	-0.07396472
N	-1.13360093	0.85797148	-1.36711581
N	-1.13394933	0.73450172	1.43065482
C	-0.72501411	-2.39122907	-1.29677797
C	-0.77452698	2.30787173	-1.41412714
C	-0.75075621	0.06009318	2.70791408

H	-0.83938982	-2.22060767	0.70337613
H	-0.85816006	0.49184086	-2.27493629
H	-0.87760420	1.70978598	1.56296904
H	3.12974394	-1.59476045	-1.39912544
H	2.91804656	-0.02375893	-2.15818104
H	3.09225982	2.06895109	-0.71938459
H	2.91540283	1.93254080	1.01308290
H	3.10925013	-0.35931875	2.11157093
H	2.95184601	-1.79616951	1.11625211
H	1.27006684	-2.81407177	-0.58394624
H	1.13770794	-2.76576089	-2.33701263
H	1.21733521	1.93796794	-2.15898697
H	1.07222130	3.42490158	-1.23045304
H	1.22948061	0.91927334	2.73888248
H	1.11334167	-0.62816299	3.56826248
H	-3.09215671	-2.06805631	-0.72162218
H	-2.91581822	-1.93354921	1.01104246
H	-3.12959527	1.59628838	-1.39748835
H	-2.91839577	0.02602063	-2.15817554
H	-3.10924638	0.35753316	2.11190417
H	-2.95178978	1.79525529	1.11783923
H	-1.21706751	-1.93689273	-2.16029664
H	-1.07187985	-3.42426512	-1.23247883
H	-1.26979689	2.81470069	-0.58239980
H	-1.13737397	2.76723954	-2.33549783
H	-1.22950315	-0.92172175	2.73802265
H	-1.11334582	0.62493688	3.56883774

Table S14. Computed coordinates of $[Co(dinosar)]Cl_3$ (**6**) with fixed $Co–N_6$ bond distances from previous 35 °C EXAFS data (1.9751(6) Å). Total energy: -2627.10776777 Hartrees

Symbol	x	y	z
Co	-0.00000612	0.00000743	-0.00319053
N	4.58211821	-0.00326077	0.00598911
O	5.15304418	1.05052806	0.11583316
O	5.06331305	-1.10648798	-0.07918376
C	3.04333872	0.03611602	-0.00691795
C	2.61839494	-0.64133181	-1.30653270
C	2.60626551	1.49363499	0.06180545
C	2.62152785	-0.76058757	1.22665161

N	1.13649010	-0.86067920	-1.37016453
N	1.11506477	1.62865567	-0.07491884
N	1.13684280	-0.73584421	1.43455772
C	0.77509870	-2.30985870	-1.41536941
C	0.72545590	2.39331978	-1.29802657
C	0.75099071	-0.06083911	2.71046686
H	0.86140310	-0.49415796	-2.27796724
H	0.84245268	2.22450934	0.70274097
H	0.88106090	-1.71141438	1.56605993
N	-4.58218430	0.00337833	0.00588206
O	-5.15317880	-1.05044671	0.11499076
O	-5.06339996	1.10667860	-0.07817561
C	-3.04337780	-0.03602482	-0.00701109
C	-2.60634379	-1.49360059	0.06076777
C	-2.61841027	0.64220504	-1.30620935
C	-2.62152625	0.75994577	1.22702928
N	-1.11509634	-1.62859625	-0.07562657
N	-1.13646107	0.86127552	-1.36983260
N	-1.13684693	0.73508376	1.43496070
C	-0.72521615	-2.39296436	-1.29883163
C	-0.77485213	2.31040716	-1.41472823
C	-0.75103082	0.05932112	2.71048118
H	-0.84269916	-2.22461432	0.70198013
H	-0.86147082	0.49485943	-2.27770459
H	-0.88105282	1.71057068	1.56706267
H	3.13220560	-1.59905387	-1.39409634
H	2.92286240	-0.03030540	-2.15820039
H	3.09462481	2.06676982	-0.72597525
H	2.92015970	1.93608966	1.00704590
H	3.11161604	-0.35254731	2.11284753
H	2.95650523	-1.79267337	1.12199629
H	1.26864745	-2.81614702	-0.58226712
H	1.13849478	-2.77130135	-2.33553722
H	1.21582557	1.93742587	-2.16170709
H	1.07282412	3.42638233	-1.23618597
H	1.22793556	0.92189163	2.73990308
H	1.11394474	-0.62392368	3.57244233
H	-3.09448797	-2.06612334	-0.72758787
H	-2.92053892	-1.93675984	1.00557874
H	-3.13201112	1.60009967	-1.39306733
H	-2.92310317	0.03183171	-2.15826712

H	-3.11163359	0.35141729	2.11299035
H	-2.95648316	1.79209579	1.12294827
H	-1.21552182	-1.93691298	-2.16247064
H	-1.07249171	-3.42607502	-1.23727954
H	-1.26838768	2.81656198	-0.58153228
H	-1.13813172	2.77209770	-2.33481670
H	-1.22799087	-0.92342021	2.73933618
H	-1.11400193	0.62190321	3.57277774

Table S15. Computed coordinates of [Co(dinosar)]Cl_3 (**6**) with fixed Co–N_6 bond distances from previous 57 °C EXAFS data (1.9776(6) Å). Total energy: -2672.10799293 Hartrees

Symbol	x	y	z
Co	-0.00001838	0.00000776	-0.00312521
N	4.58288555	-0.00331392	0.00594435
O	5.15406833	1.05055231	0.11366243
O	5.06419398	-1.10664168	-0.07729689
C	3.04383231	0.03613041	-0.00692978
C	2.61957848	-0.64336284	-1.30593766
C	2.60745161	1.49416036	0.05961782
C	2.62268804	-0.75896305	1.22815269
N	1.13790406	-0.86234714	-1.37147456
N	1.11646168	1.63068351	-0.07573622
N	1.13827720	-0.73607695	1.43679351
C	0.77520038	-2.31111593	-1.41552823
C	0.72550021	2.39409203	-1.29905635
C	0.75111186	-0.06026516	2.71174534
H	0.86318016	-0.49563355	-2.27926306
H	0.84428040	2.22646413	0.70207707
H	0.88289926	-1.71172194	1.56820770
N	-4.58289886	0.00341114	0.00593909
O	-5.15407668	-1.05052886	0.11288972
O	-5.06414609	1.10681847	-0.07650532
C	-3.04386031	-0.03605605	-0.00695980
C	-2.60753431	-1.49413878	0.05868101
C	-2.61962982	0.64419598	-1.30557160
C	-2.62269317	0.75833118	1.22857381
N	-1.11651126	-1.63062741	-0.07644661
N	-1.13792386	0.86296786	-1.37110707
N	-1.13828492	0.73532148	1.43721033

C	-0.72534746	-2.39368070	-1.29992163
C	-0.77504427	2.31170076	-1.41478088
C	-0.75113095	0.05877089	2.71177473
H	-0.84449304	-2.22660888	0.70126776
H	-0.86329537	0.49642190	-2.27899006
H	-0.88289302	1.71088573	1.56920077
H	3.13308072	-1.60144857	-1.39130119
H	2.92541075	-0.03387411	-2.15822644
H	3.09545543	2.06549461	-0.72969440
H	2.92275182	1.93803468	1.00371822
H	3.11251021	-0.34882001	2.11353614
H	2.95895309	-1.79079113	1.12508690
H	1.26794634	-2.81698353	-0.58168449
H	1.13881397	-2.77364955	-2.33507673
H	1.21505773	1.93730196	-2.16273541
H	1.07307525	3.42717518	-1.23851548
H	1.22721987	0.92289793	2.74043814
H	1.11428471	-0.62223964	3.57436861
H	-3.09538774	-2.06489746	-0.73114050
H	-2.92306484	-1.93866817	1.00239653
H	-3.13297595	1.60242379	-1.39026430
H	-2.92565354	0.03531197	-2.15822566
H	-3.11252240	0.34770797	2.11373132
H	-2.95894588	1.79021942	1.12607005
H	-1.21486828	-1.93668319	-2.16351652
H	-1.07284311	-3.42680919	-1.23970482
H	-1.26776388	2.81738185	-0.58080478
H	-1.13857850	2.77452152	-2.33421533
H	-1.22725231	-0.92440271	2.73990617
H	-1.11430463	0.62025296	3.57471845

References

1. Krause, R.; Megargel, E. Student synthesis of tris(ethylenediamine)cobalt(III) chloride | Journal of Chemical Education. *J. Chem. Educ.* **1976**, *53*, 667, doi:10.1021/ed053p667.
2. Bailar, J.C.; Work, J.B. Some Coördination Compounds of Cobalt Containing Trimethylenediamine and Neopentanediamine. *J. Am. Chem. Soc.* **1946**, *68*, 232–235, doi:10.1021/ja01206a024.
3. Geue, R.J.; Snow, M.R. Structure, conformational analysis and optical activity of a bis(tridentate)cobalt(III) complex. (+)589-Δλλ-Bis[1,1,1-tris(aminomethyl)ethane]cobalt(III) chloride (+)589-(R,R)-tartrate hydrate. *Inorg. Chem.* **1977**, *16*, 231–241, doi:10.1021/ic50168a004.
4. Qin, C.-J.; James, L.; Chartres, J.D.; Alcock, L.J.; Davis, K.J.; Willis, A.C.; Sargeson, A.M.; Bernhardt, P.V.; Ralph, S.F. An Unusually Flexible Expanded Hexaamine Cage and Its CuII Complexes: Variable Coordination Modes and Incomplete Encapsulation. *Inorg. Chem.* **2011**, *50*, 9131–9140, doi:10.1021/ic201326d.
5. Bottomley, G.; Clark, I.; Creaser, I.; Engelhardt, L.; Geue, R.; Hagen, K.; Harrowfield, J.; Lawrance, G.; Lay, P.; Sargeson, A.; et al. The Synthesis and Structure of Encapsulating Ligands: Properties of Bicyclic Hexamines. *Aust. J. Chem.* **1994**, *47*, 143, doi:10.1071/CH9940143.
6. Ozvat, T.M.; Peña, M.E.; Zadrozny, J.M. Influence of ligand encapsulation on cobalt-59 chemical-shift thermometry. *Chem. Sci.* **2019**, *10*, 6727–6734, doi:10.1039/C9SC01689A.
7. The MathWorks Inc. *Signal Processing Toolbox References*; Natick, MA, 2020;
8. Frisch, M.J.; Trucks, G.W.; Schlegel, H.B.; Scuseria, G.E.; Robb, M.A.; Cheeseman, J.R.; Scalmani, G.; Barone, V.; Petersson, G.A.; Nakatsuji, H.; et al. *Gaussian 16 Rev. C.01*; Wallingford, CT, 2016;
9. Neese, F. A spectroscopy oriented configuration interaction procedure. *The Journal of Chemical Physics* **2003**, *119*, 9428–9443, doi:10.1063/1.1615956.
10. Grimme, S.; Antony, J.; Ehrlich, S.; Krieg, H. A consistent and accurate ab initio parametrization of density functional dispersion correction (DFT-D) for the 94 elements H-Pu. *J. Chem. Phys.* **2010**, *132*, 154104, doi:10.1063/1.3382344.
11. Krishnan, R.; Binkley, J.S.; Seeger, R.; Pople, J.A. Self-consistent molecular orbital methods. XX. A basis set for correlated wave functions. *The Journal of Chemical Physics* **1980**, *72*, 650–654, doi:10.1063/1.438955.
12. Neese, F. The ORCA program system. *WIREs Computational Molecular Science* **2012**, *2*, 73–78, doi:10.1002/wcms.81.
13. Sudmeier, J.L.; Anderson, S.E.; Frye, J.S. Calculation of Nuclear Spin Relaxation Times. *Concepts in Magnetic Resonance* **1990**, *2*, 197–212, doi:10.1002/cmr.1820020403.
14. Farrar, T.; Becker, E. *Pulse Fourier Transform NMR*; Academic Press: New York, 1971;
15. The MathWorks Inc. *Curve Fitting Toolbox User's Guide R*; Natick, MA, 2020;

16. The MathWorks Inc. *Symbolic Math Toolbox*; Natick, MA, 2020;
17. Ozvat, T.M.; Sterbinsky, G.E.; Campanella, A.J.; Rappé, A.K.; Zadrozny, J.M. EXAFS investigations of temperature-dependent structure in cobalt-59 molecular NMR thermometers. *Dalton Trans.* **2020**, doi:10.1039/D0DT01391A.
